# Human antibodies targeting a *Mycobacterium* transporter protein mediate protection against tuberculosis

**DOI:** 10.1038/s41467-021-20930-0

**Published:** 2021-01-27

**Authors:** Avia Watson, Hao Li, Bingting Ma, Ronen Weiss, Daniele Bendayan, Lilach Abramovitz, Noam Ben-Shalom, Michael Mor, Erica Pinko, Michal Bar Oz, Zhenqi Wang, Fengjiao Du, Yu Lu, Jan Rybniker, Rony Dahan, Hairong Huang, Daniel Barkan, Ye Xiang, Babak Javid, Natalia T. Freund

**Affiliations:** 1 Department of Clinical Microbiology and Immunology, Sackler Faculty of Medicine, Tel Aviv University, Tel Aviv-Yafo, Israel; 2 Centre for Global Health and Infectious Diseases, Collaborative Innovation Centre for the Diagnosis and Treatment of Infectious Diseases, Tsinghua University School of Medicine, Beijing, China; 3 College of Veterinary Medicine, China Agricultural University, Beijing, China; 4 Advanced Innovation Center for Structural Biology & Beijing Frontier Research Center for Biological Structure, Tsinghua University School of Medicine, Beijing, China; 5 Pulmonary and Tuberculosis Department, Shmuel Harofe Hospital, Be’er Ya’akov, Israel; 6 Koret School of Veterinary Medicine, The Robert H. Smith Faculty of Agriculture, Food and Environment, The Hebrew University of Jerusalem, Rehovot, Israel; 7 Beijing Key Laboratory of Drug Resistance Tuberculosis Research, Department of Pharmacology, Beijing Tuberculosis and Thoracic Tumor Research Institute, Beijing Chest Hospital, Capital Medical University, Beijing, China; 8 Department of Internal Medicine, Division of Infectious Diseases, University of Cologne, Cologne, Germany; 9 German Center for Infection Research (DZIF), Bonn-Cologne, Germany; 10 Department of Immunology, Weizmann Institute of Science, Rehovot, Israel; 11 National Clinical Laboratory on Tuberculosis, Beijing Key Laboratory for Drug Resistant Tuberculosis Research, Beijing Chest Hospital, Capital Medical University, Beijing Tuberculosis and Thoracic Tumor Institute, Beijing, China; 12 Division of Experimental Medicine, University of California, San Francisco, CA USA

**Keywords:** Antibodies, Antimicrobial responses, Tuberculosis, X-ray crystallography

## Abstract

*Mycobacterium tuberculosis* (Mtb) exposure drives antibody responses, but whether patients with active tuberculosis elicit protective antibodies, and against which antigens, is still unclear. Here we generate monoclonal antibodies from memory B cells of one patient to investigate the B cell responses during active infection. The antibodies, members of four distinct B cell clones, are directed against the Mtb phosphate transporter subunit PstS1. Antibodies p4-36 and p4-163 reduce *Mycobacterium bovis-*BCG and Mtb levels in an ex vivo human whole blood growth inhibition assay in an FcR-dependent manner; meanwhile, germline versions of p4-36 and p4-163 do not bind Mtb. Crystal structures of p4-36 and p4-170, complexed to PstS1, are determined at 2.1 Å and 2.4 Å resolution, respectively, to reveal two distinctive PstS1 epitopes. Lastly, a prophylactic p4-36 and p4-163 treatment in Mtb-infected Balb/c mice reduces bacterial lung burden by 50%. Our study shows that inhibitory anti-PstS1 B cell responses arise during active tuberculosis.

## Abstract (web summary)

Antibody responses against Mycobacteria infection have been reported, but whether and how they impact anti-bacteria immunity in the host is unclear. Here the authors characterize human anti-Mycobacteria antibodies to find them targeting a Mycobacteria transporter protein, PstS1, show distinct interaction modes in crystal structure, and mediate protection in vitro.

## Introduction

Exposure to *Mycobacterium tuberculosis* (Mtb) results in a spectrum of outcomes, one of which results in active tuberculosis (ATB) disease^1^. Both innate and adaptive arms of the immune response have been implicated in immunity to Mtb^2,3^, but the role of humoral immunity and more specifically, antibodies, remains controversial^4,5^. Several studies have suggested that antibodies may play a protective role in at least a proportion of otherwise healthy individuals who have a history of exposure to Mtb^4,6–8^, and antibody responses have been correlated with the protective efficacy of an experimental TB vaccine^9^. During active disease, higher antibody titers to the mycobacterial glycolipid lipoarabinomannan (LAM) have been correlated with decreased severity of infection^10^, and there is evidence of B cell dysfunction during active disease that resolves following treatment^11^. In addition, during an active infection, serum antibodies against the Mtb phosphate transporter PstS1 are detected^12,13^. However, functional characterization of monoclonal antibodies isolated from patients with ATB has not been carried out, and it is not clear whether such antibodies exhibit any antibacterial effects.

In the present study, we explore B cell responses during ATB, while focusing on the immune-dominant antigen PstS1. We isolate and analyze a total of 85 monoclonal antibodies (mAbs) from one patient with elevated anti-PstS1 responses and find two antibodies, p4-36 and p4-163, from distinct clones that demonstrate inhibitory activity against both BCG and Mtb. Structural analysis reveals that the two antibodies are directed against two different epitopes on PstS1 and do not compete with one another. When administered prior to infection of Balb/c mice with Mtb, both antibodies cause a 50% reduction in lung bacterial burden. Our data show that both p4-36 and p4-163 exhibit antibacterial activity and provide the first proof of concept that protective antibody responses can be generated during the course of active tuberculosis disease, a finding that may inform both therapeutic and preventive vaccine design.

## Results

### Isolation of PstS1-specific mAbs

**Fig. 1:**
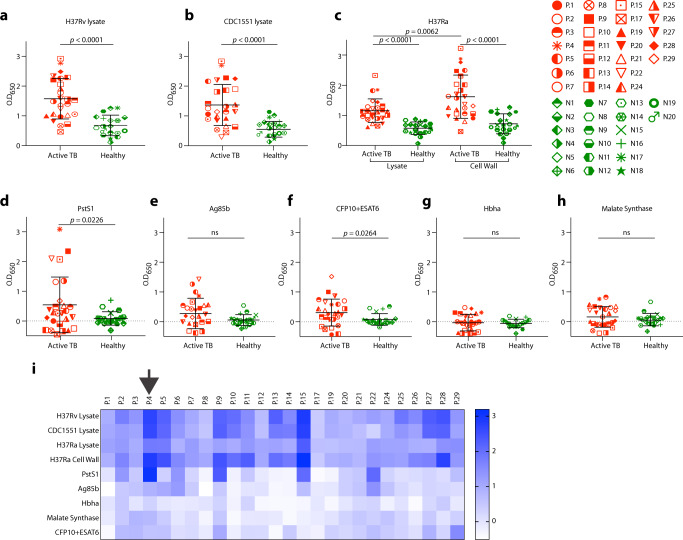
Serological profile of 26 Israeli actively infected tuberculosis patients. Serum responses by ELISA against **a** Mtb-H37Rv lysate, **b** Mtb-CDC1551 lysate, **c** Mtb-H37Ra lysate and cell-wall fraction, and recombinant proteins **d** PstS1, **e** antigen 85b (Ag85b), **f** CFP10 + ESAT6 complex, **g** Hbha, and **h** Malate Synthase. Every symbol represents a single patient, with the legend given on the right side of the figure. ATB patients are in red symbols and negative community controls are in green symbols. A heat map summarizing ELISA signals against the different antigens and lysates is shown in (**i**). Donor P.4 is marked with a black arrow. All error bars are represented as mean ± SD. All statistics were derived for *n* = 26 ATB patients versus *n* = 20 negative community controls, except for (**a**, **b**) *n* = 19 negative community controls. Significance was determined using a two-tailed unpaired Welch’s *t* test. ns no significance. Data are representative of at least two independent experiments.

We recruited 26 in-patients with ATB (Supplementary Table 1). All patients received standard treatment, recovered, and were eventually discharged. We screened patient sera for anti-Mtb antibody responses prior to initiation of antibiotics using lysates from two pathogenic Mtb strains, H37Rv and CDC1551. Unlike healthy community controls, the majority of patients (23/26) showed serum reactivity by ELISA to at least one lysate (Fig. 1a, b). Testing of antibody titers against soluble lysate versus cell-wall components of nonpathogenic Mtb-H37Ra^14^ revealed responses against both components, but with higher reactivity against the Mtb surface (Fig. 1c). To identify antibody reactivity against specific Mtb proteins, we tested sera against five selected surface-exposed proteins (produced in-house, Supplementary Fig. 1) that had been described previously to elicit antibody responses in human infection (Fig. 1d–h)^15,16^. The strongest response was directed against PstS1^12^ (Fig. 1d, i), which has been implicated in Mtb virulence^13^ and is one of three genes previously identified to contain amino acid sequence variants undergoing diversifying selection in conserved epitopes^17,18^. Therefore, we decided to characterize antibody responses to PstS1.

**Fig. 2:**
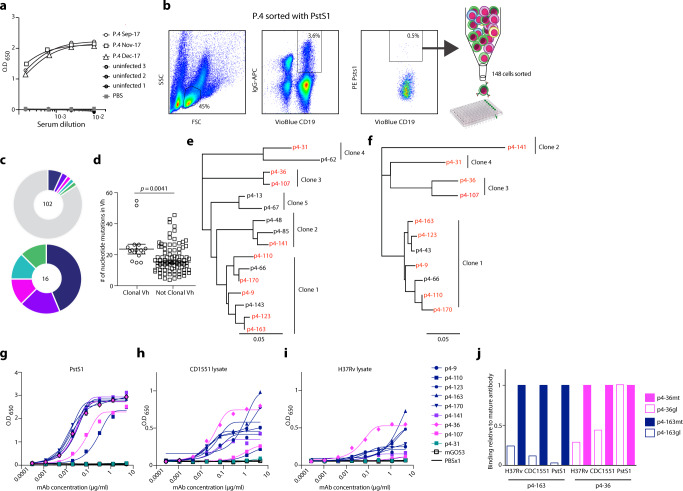
Isolation of anti-PstS1 mAbs from memory B cells of P.4. **a** Donor P.4 serum responses taken at three different time points (indicated) to recombinant PstS1 by ELISA. **b** Isolation of PstS1-specific B cells. Whole-blood-derived lymphocytes were stained for CD19, membrane IgG, and PstS1. A total of 148 positive cells were single-cell sorted. **c** Pie charts representing the heavy chain sequences that were amplified from the CD19+/IgG+/PstS1+ B cells from P.4. The number in the middle of the pies denotes the total number of sequences, and the colored slices indicate clonally related sequences. Upper panel: all sequences. Lower panel: only the 16 clonally related sequences: clone 1—dark blue, clone 2—purple, clone 3—magenta, clone 4—teal, clone 5—green. **d** Nucleotide mutations in V_H_ of the clonal versus nonclonal sequences. Error bars are represented as mean ± SEM. *n* = 16 clonal Vh sequences and n = 85 nonclonal Vh sequences. **e**, **f** are dendrograms (created using Geneious software) of the clonally related sequences, heavy and light chains, respectively. The mAbs selected for expression are colored in red. **g–i** The binding of nine mAbs by ELISA to recombinant PstS1 (**g**), Mtb-CDC1551 lysate (**h**), and Mtb-H37Rv lysate (**i**). **j** Comparing binding by ELISA of the mature (mt) and the predicted germline (gl) versions of p4-36 (magenta) and p4–163 (dark blue), to recombinant PstS1, Mtb-CDC1551 lysate, and Mtb-H37Rv lysate. AUC binding score of each antibody was determined as relative to the AUC of the mature antibody, which was normalized to 1 (see also Supplementary Fig. 5). All data are representative of at least two independent experiments (**g**–**j**).

We focused on one patient, P.4, who had the strongest individual response to PstS1 and retained high anti-PstS1 responses over his treatment course (Fig. 2a). Hence, we sought to isolate mAbs directed against PstS1 and test their effect on experimental models of Mtb infection. We isolated peripheral blood mononuclear cells (PBMCs) from whole blood and sorted single IgG+, PstS1+ B cells, which comprised 0.5% of the total IgG+ B cell population (Fig. 2b)^19^. A total of 102 heavy and 90 light chains were amplified by single-cell Ig PCR;^20^ of these, 85 constituted natural heavy and light pairs (Supplementary Fig. 2). Consistent with previous reports of anti-Mtb B cell responses in humans^7^, the majority of the antibody sequences were not clonal. Based on V_H_ D_H_ J_H_, V_L_, and J_L_ and >75% identity in CDRH3^21^, a total of five clonal families were identified in 16 sequences (Fig. 2c). As previously described^21^, the V_H_ sequences of the clonal heavy chains were more mutated when compared to the V_H_ sequences that were not part of a clone (Fig. 2d), but both groups had a similar V_H_ gene usage distribution (Supplementary Fig. 2b). We produced nine antibodies derived from four clones expressed in an IgG1 expression vector^20^ (Fig. 2e, f, expressed antibodies are in red). We were unable to amplify the light chains from Clone 5 variants and therefore antibodies from this clone were not produced. All tested mAbs, except for p4-31, reacted strongly both with PstS1 (with dissociation constants within the nanomolar range, Supplementary Fig. 3) and with Mtb lysates by ELISA (Fig. 2g–i). Among the nine antibodies we produced, mAbs p4-36 and p4-163 exhibited the strongest binding to bacterial lysates (Fig. 2h, i). These mAbs also showed binding to whole-bacteria H37Ra-mCherry compared with isotype control (Supplementary Fig. 4). We therefore decided to focus on these antibodies for the rest of the study.

First, since the anti-PstS1 mAbs were generated during natural infection occurring in patient P.4, we sought to investigate the impact of affinity maturation on their activity. Both p4-36 and p4-163 showed relatively low levels of somatic hypermutations, with p4-36 exhibiting 6 and 12 amino acid changes in heavy and light chains, respectively, and p4-163 exhibiting 10 and 8 amino acid changes in heavy and light chains, respectively. Nevertheless, we hypothesized that these mutations provide the mAbs with their ability to bind PstS1 and Mtb lysates. To test our hypothesis, we reverted the amino acid sequences of p4-36 and p4-163 back to their germline sequences (Supplementary Fig. 5a, b), similarly to what was previously carried out for other naturally elicited mAbs^22^, thus producing the unmutated predicted germline (gl) versions of each antibody: p4-36gl and p4-163gl. The binding of the germline variants was compared to the binding of the mature antibodies to PstS1, as well as H37Rv and CDC1551 Mtb lysates. Both mAbs showed a dramatic reduction in their binding when somatic hypermutations were reverted, with the exception of mAb p4-36gl that exhibited similar binding to PstS1 as the mature mAb (p4-36mt) by ELISA. However, SPR measurements showed a 4.7-fold reduction in affinity (Fig. 2j, Supplementary Fig. 5c–g). To further investigate whether mutations occurring in the heavy or the light chain contribute to antibody activity, we produced chimeric antibodies where the heavy chain is mature while the light chain is germline (HCmtLCgl), and vice versa (HCglLCmt). Testing those for binding showed that for p4-36, mutations in the light chain were more significant for binding than those in the heavy chain, as opposed to p4-163 where mutations in the heavy chain were more essential for binding compared to mutations in the light chain (Supplementary Fig. 5c–g). Overall, we conclude that both mAbs p4-36 and p4-163 are specific for PstS1 and acquired somatic hypermutations during natural infection that resulted in their improved binding to Mtb.

### Activity ex vivo

**Fig. 3:**
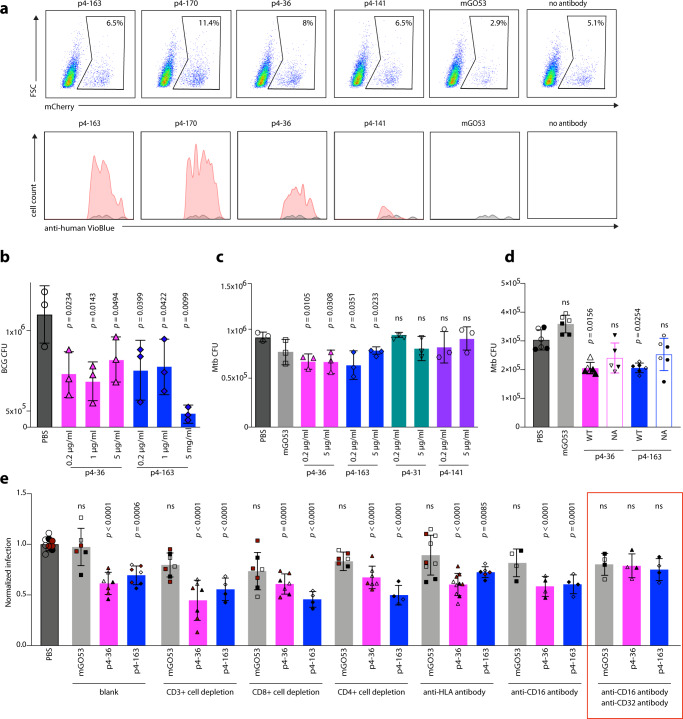
Anti-PstS1 mAbs inhibit Mtb in culture. **a** Upper panel: gating strategy of H37Ra-infected macrophages, mCherry positive. PMA-differentiated THP-1 cells preincubated with mAbs p4-163, p4-170, p4-36, p4-141, and the isotype control mAb mGO53^20^. For each treatment *n* = 90,000 cells were analyzed by flow cytometry. Lower panel: histograms showing the frequencies of intracellular antibody-bound bacteria as detected by anti-human VioBlue antibody staining. For each mAb, the binding histogram is shown in red and compared to the isotype control histogram, which is depicted in a gray overlay. **b**, **c** Activity of anti-PstS1 mAbs at indicated concentrations in a human whole blood mycobacterial growth inhibition assay (MGIA) after 96 h of infection with BCG or pathogenic Mtb, respectively. CFU was determined in *n* = 3 biological repetitions. **d** Activity of anti-PstS1 mAbs (5 µg/ml) used as IgG1 (named “WT”, full columns) or as N279A Fc variants (named “NA”, empty columns) in MGIA. Black and clear shapes correspond to two independent experiments. CFU was determined in *n* = 5–6 biological repetitions. **e** Activity of anti-PstS1 mAbs (5 µg/ml) in MGIA following depletion of CD3+ T cells, CD4+ T cells, CD8+ T cells, blockade of MHC II (anti-HLA), and CD16 or CD32 or both (anti-CD16, anti-CD32, marked with a red rectangle). Black, cayenne, and clear shapes correspond to three representative independent experiments. In each experiment, the data points were compared to average CFU infection in PBS, which was normalized to 1. CFU was determined in *n* = 4–10 biological repetitions. All error bars are represented as mean ± SD. Significance was determined using a one-tailed unpaired *t* test (**b**), two-tailed unpaired *t* test (**c**, **d**) for black shapes (**d**), or one-way ANOVA with Tukey’s multiple-comparison test (**e**). All statistical analyses are relative to PBS. ns no significance. Data are representative of at least two independent experiments.

Next, we sought to test the effects of the mAbs p4-36 and p4-163 on Mtb infection in culture. First, we tested the effect of our mAbs on bacterial entry. We infected phorbol 12-myristate 13-acetate (PMA) differentiated THP-1 macrophages^23^ with the attenuated H37Ra strain with and without PstS1-specific mAbs. The mAbs bound H37Ra within macrophages and did not prevent, but rather slightly increased, bacterial entry into the macrophages (Fig. 3a, upper panel, mAb binding is shown in the lower panel). We next asked whether the mAbs p4-36 and p4-163 could inhibit mycobacterial growth. For this, we used a whole-blood mycobacterial growth inhibition ex vivo assay (MGIA) where human whole blood cells from healthy donors are infected with pathogenic bacteria^24^. This system has been used previously to test polyclonal antibodies from healthy donors^25^ and allows testing of antibody activity in a more physiologically relevant in vitro system. Here, p4-36 and p4-163 were able to significantly restrict the growth of both *Mycobacterium bovis*-BCG (BCG) and pathogenic Mtb (Fig. 3b, c)^24,26^. This activity was not dose-dependent and was unique to mAbs p4-36 and p4-163, and was not observed with other anti-PstS1 mAbs, such as p4-31 and p4-141, showing that ELISA binding to Mtb lysates correlated well with their activity ex vivo.

We next sought to investigate the mechanism of mAb-induced CFU reduction over time. MAbs p4-36 and p4-163 did not inhibit infection, and immediately after infection, more antibody-bound bacteria were found in THP-1 cells (Fig. 3a). This suggests antibody-dependent opsonization of bacteria via Fc gamma receptors (FcγRs)^6,8,24^. To test this, we compared the antibody activity in MGIA by comparing the wild-type IgG1 antibodies with IgG1-N297A (NA), an aglycosylated Fc variant with no binding to FcγRs^27^. Treating with the NA variants abolished protection compared with wild-type IgG1 mAbs (Fig. 3d). To evaluate the contribution of FcγRs^+^ effector cells to the inhibitory activity of the antibodies p4-36 and p4-163, we repeated the MGIA experiments with selective FcγR blockers. Blocking the main FcγRs expressed by human macrophages, CD16 (FcγRIIIA) and CD32a, and CD32b (FcγRIIA and FcγRIIB) together eliminated the inhibitory activity of both p4-36 and p4-163 (Fig. 3e). By contrast, specific depletion of T cells or blocking MHC class II molecules did not affect mAb-induced CFU reduction (Fig. 3e). Altogether, our data imply that the Fc domain of the antibodies facilitates their antibacterial activity. We suggest that FcγRs can mediate bacterial opsonization into the cells, thus promoting the antibody inhibition of infection. Whether this Mtb inhibition is due to an active restriction of intracellular bacterial replication or due to induction of bacterial killing is yet to be determined.

### Structure–function studies

**Fig. 4:**
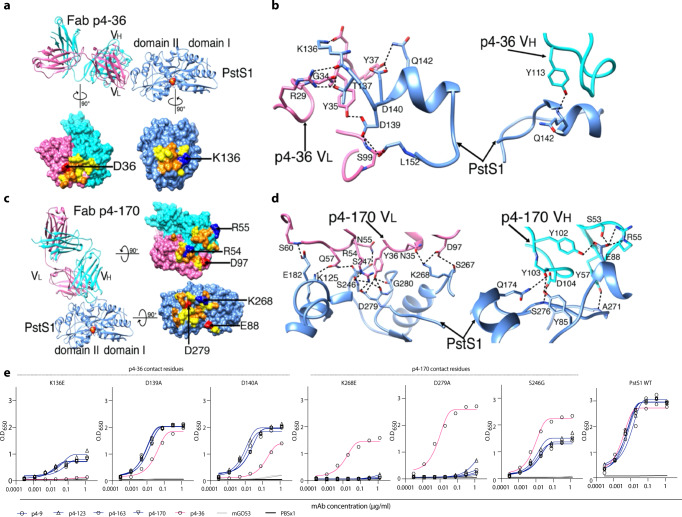
MAbs p4–170 and p4–36 recognize different epitopes on top of PstS1. **a** Top: Ribbon diagrams show the crystal structure of PstS1 in a complex with Fab p4–36 (PDB ID 7DM1). The Fab p4-36 heavy and light chains are colored cornflower cyan and hot pink, respectively. The PstS1 structure is colored blue. The bound phosphate (Pi) is represented as filled balls with oxygen and phosphorus atoms colored red and yellow, respectively. Bottom: an open-up surface-shadowed representation showing the contact interface. Residues involved in hydrogen bonds and Van der Waals contacts are highlighted in orange and yellow, respectively. Positively and negatively charged residues involved in the formation of the salt bridges are highlighted in blue and red, respectively. **b** Close-up view of the interface between PstS1 and Fab p4-36. The dashed lines indicate hydrogen bonds and salt bridges. **c** Left: Ribbon diagrams show the crystal structure of PstS1 in complex with Fab p4-170 (PDB ID 7DM2). Right: An open-up surface-shadowed representation showing the contact interface. The color schemes used are the same as in (**a**). **d** Close-up view of the interface between PstS1 and Fab p4-170. The dashed lines indicate hydrogen bonds and salt bridges. **e** Four Clone 1 variants (mAbs p4-9, p4-123, p4-163, and p4-170, dark blue), and mAb p4-36 (magenta), as well as negative control mAb mGO53^20^ (gray), were tested for binding by ELISA to six-point mutant PstS1 proteins. The binding curves of PstS1 mutated in p4-170 contact residues K268E, D279A, and S246G, as well as the PstS1-mutated p4-36 contact residues K136E, D139A, and D140A are shown. The binding curves to wild-type PstS1 are shown in the right panel of the figure.

To further understand the mechanism of the protective antibody binding to its target, we produced antigen-binding fragments (Fabs) of p4-163 and p4-36 and prepared Fab–PstS1 complexes for crystallization. We determined the structure of Fab p4-36 in complex with PstS1 at a resolution of 2.1 Å (Fig. 4a and Supplementary Table 2). Two PstS1–Fab p4-36 heterodimers were in the asymmetric unit of the crystal and had only minor differences in the constant domains of the bound Fabs (Supplementary Fig. 6). P4-36 binds a contiguous epitope located on an alpha-helix formed by residues 141–145 and preceding residues 136–137 and residues 139–140 (Fig. 4b and Supplementary Fig. 7a), within a small surface area of 583 Å^2^. The contacts between p4-36 and PstS1 are mostly Van der Waals and hydrogen bonds and contributed largely by complementarity-determining region 1 (CDR1) and CDR3 of the light chain (CDRL1 and L3) and CDR3 of the heavy chain (CDRH3) (Fig. 4b, Supplementary Fig. 8a, b and Supplementary Table 3). Ten hydrogen bonds are observed at the interface, and one salt bridge is formed between Asp36^p4–36 CDRL1^ and Lys136^PstS1^ (D36^L^[OD1]-K136^PstS1^[NZ]) (Fig.4b and Supplementary Table 3).

No crystal could be obtained with the complex of Fab p4-163 and PstS1. Thus, we switched to p4-170, which is a closely related variant of p4-163 and a member of the same B cell clone (Clone 1, Fig. 2e, f), and has 97.6% and 93.86% amino acid sequence identity to p4-163 heavy and light chains, respectively (Supplementary Fig. 5a, b). In addition, p4-170 exhibited similar inhibitory activity in MGIA to p4-163 (Supplementary Fig. 9). The Fab p4-170 and PstS1 complex could be crystallized, and the structure was determined at a resolution of 2.4 Å with one PstS1–Fab p4-170 heterodimer in the asymmetric unit (Fig. 4c and Supplementary Table 2). As revealed by the structure of the complexes, p4-170 binds to a different epitope from the one recognized by p4-36.

The epitope recognized by p4-170 is highly discontinuous and conformationally proximate to the Pi binding site in the middle of the molecule, between the two domains I and II of PstS1 (Fig. 4c). The epitope is a large surface of 977 Å^2^ and comprises three helices and four loops of PstS1 (Fig. 4d and Supplementary Fig. 7b). The interactions between p4-170 and PstS1 are mediated through salt bridges, hydrogen bonds, and Van der Waals contacts (Fig. 4d and Supplementary Table 4). Except for CDRH1, all the CDRs of p4-170 are involved in the interactions with residues 85, 88, and 92 of the PstS1 domain I and residues 125, 174, 177–178, 181–182, 190, 246–248, 267–268, 270–271, 275–276, and 279–281 of domain II (Fig. 4d, Supplementary Fig. 8c, d and Supplementary Table 4). The hydrogen bonds are formed with both the main-chain and side-chain atoms of PstS1. Four salt bridges are formed at the contact interface, including Glu88^PstS1^[OE1]–Arg55^H^[NE], GLu88^PstS1^[OE1]–Arg55^H^[NH1], Lys268^PstS1^[NZ]–Asp97^L^[OD1], and Asp279^PstS1^[OD2]–Arg54^L^[NH2] (Fig. 4c, d and Supplementary Table 4).

To confirm the key antibody: antigen contact residues identified by the structures, we generated a panel of point mutations in PstS1. Mutating residues at the p4-170:PstS1 interface, S246G^PstS1^, K268E^PstS1^, and D279A^PstS1^ reduced the binding of all tested Clone 1 mAb variants, but not the binding of p4-36 (Fig. 4e). Amino acid substitutions in the alpha-helix that holds the epitope of p4-36 residues K136E^PstS1^, D139A^PstS1^, and D140A^PstS1^ reduced p4-36 binding. The binding of Clone 1 mAbs was also reduced, indicating that the alpha-helix bound by p4-36 might be essential for the folding of PstS1.

**Fig. 5:**
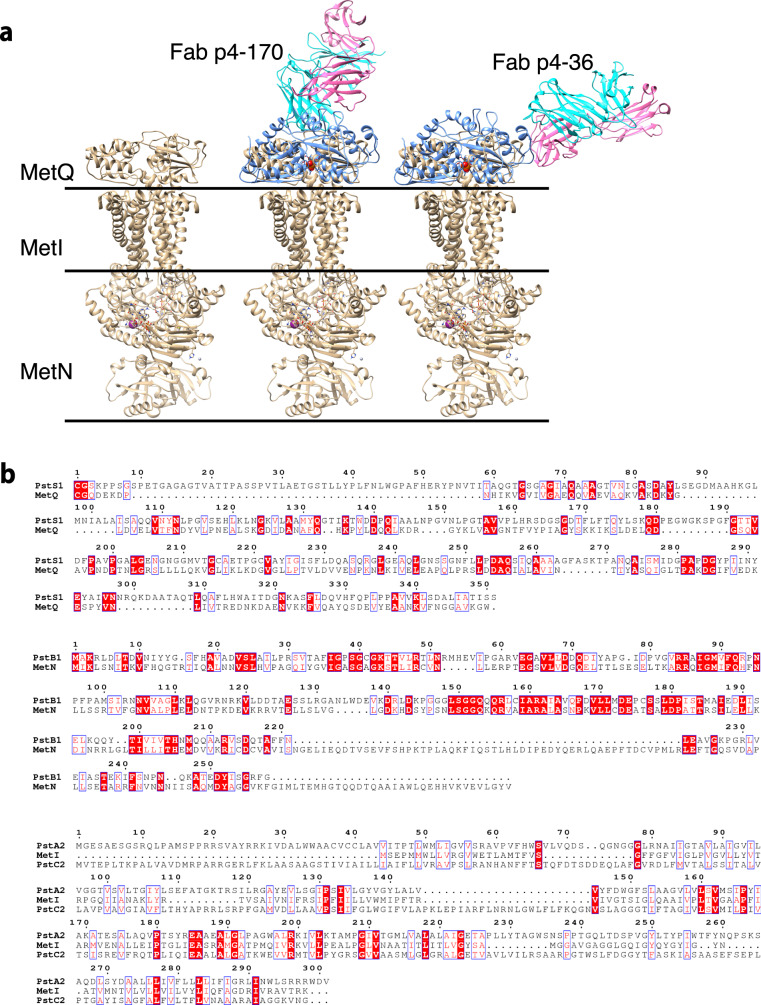
Structure modeling of the binding of PstS1 to the PstA–B–C phosphate transporter complex. **a** Structural superimposition of the Fab–PstS1 complexes with MetQ of the MetNIQ ABC transporter complex (in gold) (PDB code: 6CVL) suggested a possible binding mode of PstS1 to the PstA–B–C phosphate transporter complex. The bound antibodies show no blockage to the assembly of the PstA–B–C–S complex. **b** Sequence alignments showing that the MetNIQ complex components share significant sequence similarities to these of the PstA–B–C phosphate transporter complex of Mtb.

We next asked whether the binding of p4-36 or p4-170 could interfere with multimerization of the transporter. For this, we performed modeling of the binding of PstS1 to the transporter complex. According to our prediction, the bound antibodies show no blockage to the assembly of the PstA–B–C–S complex and do not block the binding of PstS1 to the PstA, B, C transporter complex (Fig.  5). We conclude that p4-36 and p4-170 do not function by inhibiting the transporter activities .

### Activity in vivo

**Fig. 6:**
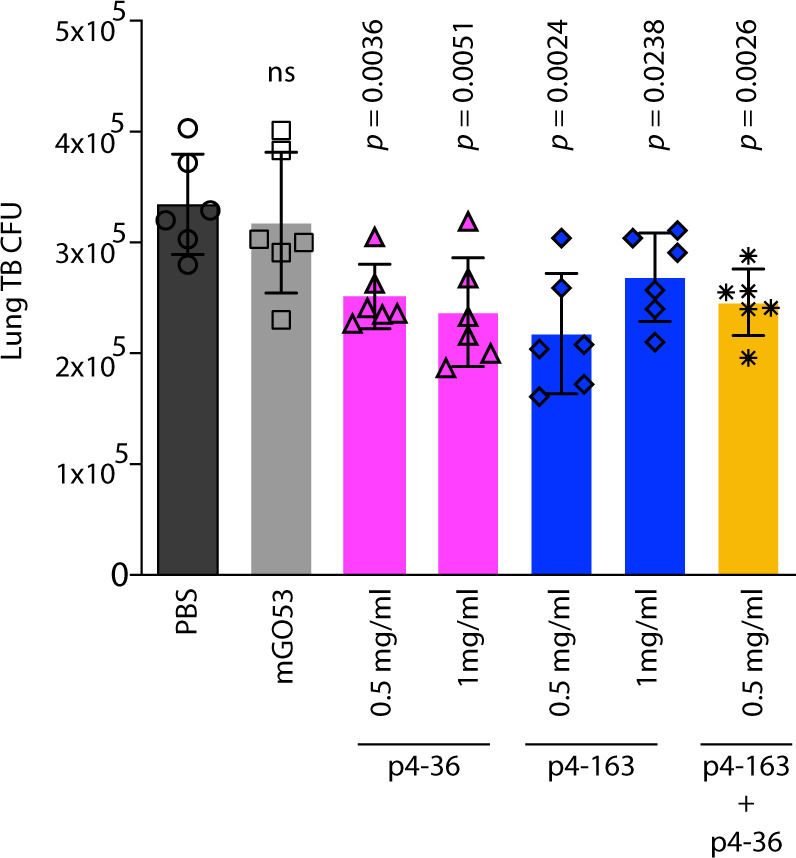
Anti-PstS1 mAbs inhibit Mtb in Balb/c mice. Activity of anti-PstS1 mAbs: p4-36 (magenta bars), p4-163 (dark-blue bars), as well as the negative control mAb mGO53 (gray bars) at indicated concentrations in Balb/c mice. Mice were injected once intraperitoneally with mAbs at indicated amounts 5 hours prior to aerosol infection with pathogenic Mtb. Mice were euthanized after 2 weeks, and lung bacterial burden measured. Error bars are represented as mean±SD. In each treatment *n* = 6 mice. Significance was determined using a two-tailed unpaired *t* test. ns no significance. The results are representative of three independent experiments.

Finally, we tested the activity of the two mAbs p4-36 and p4-163 on Mtb infection in vivo. For this, we used wild-type Balb/c mice. We injected 0.5 or 1 mg mAb per mouse intraperitoneally five hours prior to aerosol infection with pathogenic Mtb. After 2 weeks, the mice were sacrificed and lung bacterial burden determined. Lung burdens were reduced in mice pretreated with mAbs p4-36 and p4-163, or both by approximately 0.5 log CFU (Fig. 6), which agrees with the MGIA results and verifying that the mAbs have anti-Mtb-inhibiting activity.

## Discussion

Here, we describe a patient, P.4, who had active tuberculosis and was selected from a cohort of infected patients due to his strong anti-Mtb serum responses to the known immunodominant antigen PstS1. While the activity of the two anti-PstS1 mAbs we isolated is modest (about 0.5 log), to our knowledge, this is the first description that naturally elicited PstS1 antibodies have anti-Mtb activity in infection.

PstS1 is a 38-kDa phosphate-binding periplasmatic protein, one of three subunits of the Mtb phosphate-specific transporter (Pst) complex^12^. PstS1 is an immunodominant marker for ATB^28–30^. PstS1 is also necessary for Mtb virulence; PstS1-deletion mutants were attenuated in a mouse-infection model^13^. Intriguingly, PstS1 was identified as one of only three Mtb genes that are subjected to evolutionary sequence diversification, suggesting that PstS1 variation plays a role in Mtb immune evasion^17,18^.

In the present study, we characterize two different sites on PstS1 that can be targeted by antibodies capable of inhibiting Mtb growth. P4-163 is a member of the largest B cell clone of nine mAbs that shared >93% sequence identity. The high-resolution structure of a clonal variant of p4-163, p4-170, revealed a large, sparse, and highly conformational epitope that is adjacent to the active site of PstS1. Intriguingly, seven of p4-170 contact residues (Lys268^PstS1^, Pro270^PstS1^, Ala271^PstS1^, Ile275^PstS1^, Ser276^PstS1^, Asp279^PstS1^, and GLy280^PstS1^) overlap with a highly conserved Mtb T-cell epitope 259-AAAGFASKTPANQAISMIDG-280, Immune Epitope Database number 35^17,31^. The mAb p4-36, a member of a different and smaller B cell clone, binds an alpha-helix structure within residues 136^PstS1^–145^PstS1^, with the epitope being 30 Å distant from the epitope bound by p4-170.

Donor P.4 had active tuberculosis; therefore, by definition, the mAbs we isolated did not prevent or eliminate Mtb infection in this patient. However, evidence from other infectious diseases shows that B cell responses develop in parallel and subsequent to high pathogen loads that stimulate B cell activation and maturation, causing neutralizing/protective antibodies to arrive ‘too little, too late', without a significant benefit to the individual that produced them^22,32–34^. Considering the low extent of affinity maturation, as reflected by the relatively low number of somatic hypermutations in p4-36 and p4-163, it is possible that these B cell clones only started their evolution and did not reach their full anti-Mtb potential. On the other hand, the fact that these antibodies ultimately arise provides evidence that B cells play an active role during ATB.

Despite the long-standing controversy, recent studies have demonstrated that in certain cases, anti-Mtb humoral responses can be protective^7,24,35^ or correlate with lack of active disease or even lack of infection^4,6,35,36^. Only one prior study profiled the human B cell responses against Mtb antigens on a monoclonal level^7^. This study attributed bacterial inhibition to IgA isotypes rather than IgG. However, in that study, ‘protection’ was defined as decreased viable Mtb within cells shortly after infection. Unfortunately, the study evaluated neither in vivo activity nor the precise molecular mode of antigen binding. While the Balb/c mouse model we use in this study does not recapitulate lung granulomas, which are the hallmark of active TB infection in humans, passive transfer of mAbs before pathogenic Mtb infection resulted in a significant reduction of bacterial lung loads in mice indicating a preventive mAb activity.

This is the first report of human anti-Mtb mAbs that have a modest, yet robust, activity in vivo, as well as resolving their structure along with their corresponding Mtb target. PstS1 DNA was previously reported to elicit protective T-cell immunity against Mtb in mice^37^. The fact that the germline version of p4-36, p4-36gl, binds recombinant PstS1 suggests that PstS1 could be used as an immunogen in humans to elicit B cell responses and p4-36-like antibodies in naive populations. Our study has implications for the development of new anti-Mtb therapies and prevention.

## Methods

### Study design

The objective of this study was to investigate whether antibodies isolated during active tuberculosis (ATB) disease can inhibit Mtb in vivo and elucidate their corresponding Mtb target and mechanism of action. Donor P.4 was selected based on exceptional serum binding to Mtb lysates and to recombinant PstS1 protein. Staining with PstS1 and single-cell sorting of antigen-positive memory B cells from donor P.4 allowed isolation of four new clones of antibodies, two of which were able to inhibit the growth of pathogenic Mtb ex vivo and in vivo. We analyzed structurally the two mAbs and discovered that they are directed against two nonoverlapping sites on PstS1. Their ability to inhibit Mtb growth was tested by infecting healthy human whole blood cells in vivo, in Balb/c mice. The epitopes were identified by X-ray crystallography.

### Ethics statement

For the patient studies, the Tel Aviv University Institutional Review Board (IRB) approved all studies involving patient enrollment, sample collection, and clinical follow-up. Donor P.4 provided written informed consent prior to participating in this study, and The Tel Aviv University and Shmuel Harofe Hospital Institutional Review Boards approved all studies involving patient enrollment, sample collection, and clinical follow-up (protocol numbers 33.18 and 0058). Donor P.4 was selected from a group of 26 active pulmonary TB patients that were followed by the Pulmonary and Tuberculosis Department of Shmuel Harofe hospital in Israel and is also referred to as subject ID 109004. Fresh human whole blood for MGIA assays was obtained from volunteers under IRB-approved protocol (2014-2-25) of the Beijing Tumor and Thoracic Hospital. For the mouse studies, this study was carried out in strict accordance with the guidelines of the Chinese Association for Laboratory Animal Sciences and approved by the Animal Ethics Committee of Beijing Chest Hospital, Capital Medical University.

### Study participants

Twenty-six active pulmonary TB patients were recruited from the Pulmonary and Tuberculosis Department of Shmuel Harofe hospital in Israel. Patients were diagnosed with active pulmonary TB by sputum smear and positive bacterial culture and treated with antibiotics. Blood samples for screening for anti-Mtb serum antibodies were drawn before receiving antibiotic therapy. For patients who exhibited high anti-Mtb serum activity, the blood draw was repeated two additional times during hospitalization. Donor P.4, ID 109004 (Supplementary Table 1), a 35-year-old male, was diagnosed with drug-sensitive TB, and a small blood donation was first collected on September 25, 2017. This donor was asked for a large donation of whole blood, from which PBMCs were isolated by Ficoll gradient density centrifugation. Serum samples of healthy donors served as controls and were obtained from the Israeli blood bank. TB exposure history of these subjects was not available.

### Bacterial culture, lysate, and cell-wall fractions

Lysates of Mtb-H37Rv (Cat # NR-14822) and the Mtb-CDC1551 (Cat # NR-14823) were obtained from BEI resources https://www.beiresources.org/. For the preparation of H37Ra^14^ lysate and cell-wall fractions, H37Ra bacterial culture was grown in Middlebrook 7H9 broth supplemented with 10% OADC, 0.05% Tween-80, and 0.5% glycerol, at 37 °C in a shaking incubator to reach an optimal density of O.D_600_ = 0.4. The bacterial culture was centrifuged at 3180 *g* for 15 min at room temperature. The pellet was resuspended in phosphate-buffered saline (PBS)×1 containing 1% phenylmethylsulfonyl fluoride (Sigma) and was lysed by sonication. Following centrifugation at 29,800 *g* for 30 min at 4 °C, the lysate was clarified, and the cell-wall fraction was resuspended in PBS×1.

### Expression of recombinant Mtb antigens

Mtb antigens PtsS1 (Rv0934), Ag85b (Rv1886c), Hbha (Rv0475), CFP10 (Rv3874), ESAT6 (Rv3875), and Malate Synthase (Rv1837) were cloned into a pMALp vector as a fusion to maltose-binding protein (MBP), with a TEV cleavage site separating the recombinant Mtb proteins and MBP (Supplementary Fig. 1). The recombinant Mtb proteins were C-terminally tagged with 6×His and a specific BirA biotinylation sequence and were expressed in *Escherichia coli* (*E-coli*, BL21 strain). Shortly, plasmid-transformed *E. coli* was grown to an optimal density of O.D_600_ = 0.6., at which protein expression was induced by adding isopropyl-β-d-1-thiogalactopyranoside (IPTG) (0.5 mM), and the culture was grown for 4 h at 30 °C in a shaking incubator of 225 rpm. Next, bacteria were pelleted by centrifugation at 9700 *g* for 15 min at 4 °C, and the pellet was frozen and kept at a temperature of −20 °C. Afterward, bacterial pellets were thawed and resuspended in 50 mM NaH_2_PO_4_, 300 mM NaCl, 10 mM imidazole, pH = 8, Triton-X 0.1% (Sigma), and protease inhibitor cocktail (Sigma) (“binding buffer”), and lysed by sonication. Following additional centrifugation at 29,800 *g* for 30 min at 4 °C, the protein was purified from the supernatant phase using Ni sepharose beads (GE Healthcare), TEV cleavage, and elution with 50 mM and 100 mM imidazole (Sigma). Enzymatic site-specific biotinylation was carried out by BirA biotinylation kit (Avidity). All recombinant Mtb proteins were stored in PBS×1.

### ELISAs

High-binding 96-well ELISA plates (Corning, 9018) were used for all experiments. For sera binding to Mtb strains, plates were coated overnight at 4 °C with 1–2.5 μg/ml of pathogenic bacterial lysates, H37Rv, and CDC1551 (both received from BEI resources), or H37Ra lysate or cell-wall fractions. For sera binding to recombinant Mtb antigens, plates were coated overnight at 4 °C with 5 μg/ml of PstS1, Ag85b, Hbha, Malate Synthase, and ESAT6 + CFP10. ESAT6 + CFP10 were premixed at a 1:1 ratio. The next day, after washing 3 times with PBS + 0.05% Tween-20, the plates were blocked for 2 h at room temperature with 3% bovine serum albumin, 20 mM EDTA, and 0.05% Tween-20 in PBS (“blocking buffer”), followed by a 1-hour incubation at room temperature with polyclonal Mtb-infected sera or negative healthy controls at 1:100 and 1:300 dilutions in blocking buffer. After an additional washing step, the plates were incubated for 1 h with peroxidase-conjugated goat anti-human IgG (Jackson ImmunoResearch) at a final concentration of 0.16 μg/ml at room temperature and developed by adding TMB substrate (Abcam). The plates were read in an ELISA plate reader after 20 min at O.D_650_.

For anti-PstS1 and germline mAbs ELISA, plates were coated overnight at 4 °C with 5–10 μg/ml of recombinant PstS1 antigen or pathogenic bacterial fractions as described above. The next day, after washing and blocking as indicated above, plates were incubated for 1 h at room temperature with anti-PstS1 or germline mAbs in eight consecutive fourfold dilutions, starting from concentration of 5 μg/ml. After an additional washing step, the plates were incubated with peroxidase-conjugated goat anti-human IgG, developed, and read as indicated above.

For PstS1 mutant ELISA, plates were coated overnight at 4 °C with 5μg/ml of recombinant PstS1 wild type and mutants. The next day, after washing and blocking as indicated above, plates were incubated for 1 h at room temperature with anti-PstS1 mAbs in eight consecutive fourfold dilutions, starting from concentration of 1.25 μg/ml. After an additional washing step, the plates were incubated with peroxidase-conjugated goat anti-human IgG, developed, and read as indicated above.

### Single B cell sorting, sequencing, cloning, and expression of antibodies

Patients’ purified PBMCs were isolated by ficoll (GE Healthcare) and frozen at liquid nitrogen. PBMCs were thawed and enriched for B cells by CD20 magnetic microbeads (MACS, Miltenyi Biotec). The CD20+ B cell fraction was stained for 15 min at 4 °C with anti-human antibodies: CD19-VioBlue (1:100, Miltenyi Biotec), IgG-APC (1:20, Miltenyi Biotec), and with biotinylated PstS1 preincubated with streptavidin-PE (1:10, Miltenyi Biotec). All the PstS1-PE-positive IgG+ CD19+ cells were single-cell sorted into 4 μl of lysis buffer (PBS, RNAsin-Promega, and 0.1 M DTT). Rescue primers were used to amplify both heavy chains^38^ and Igλ genes^39^, and regular primers were used for IgK chains^40^. All PCR products were sequenced and analyzed for Ig gene usage, CDR3, and the number of V_H_/V_L_ somatic hypermutations (IgBLAST, www.ncbi.nlm.nih.gov/igblast and IMGT, www.imgt.org). Purified, digested PCR products were cloned into human Igγ1, Igk, or Igλ-expression vectors as previously described^40^ and produced by transient transfection of IgH, IgK, and IgL expression plasmids into exponentially growing Expi293F cells, as previously described^41^.

### Biacore (SPR)

Biocare assay was performed on a Biacore T200 instrument at 25 °C. In total, 5 μg/ml Capture Select^TM^ Biotin Anti-IgG-Fc (multispecies) Conjugate (ThermoFisher Scientific) was immobilized to sensor chip SA, series S (GE Healthcare) with a contact time of 600 s and flow rate of 10 μl/min. In total, 0.25 μg/ml anti-PStS1 mAbs were injected with a contact time of 600 s and a flow rate of 10 μl/min. Following anti-PStS1 mAbs binding to the Capture Select, PstS1 was injected in four concentrations: 7.81, 31.25, 125, and 500 nM. Following each cycle, the regeneration step was done with 0.1 M glycine, pH 2 at a flow rate of 30 μl/min for 1.5 min. Three replicates were performed for each mAb and all samples were diluted in HBS-EP buffer (0.01 M HEPES, 0.15 M NaCl, 0.003 M EDTA, and 0.05% Tween-20, pH 7.4). Sensograms were fitted to a 1:1 binding model using nonlinear regression in the BIA evaluation software. *K*_D_ was calculated using the ratio of the kinetic constants *K*_D_ = *K*_d_/*K*_a_.

### Flow cytometry for direct and intracellular antibody binding

The binding of p4-mAbs to H37Ra-mCherry was determined as follows: bacteria were grown to OD_600_ = 0.4, centrifuged, and washed with PBS. A total of 10^8^ colony-forming units (CFU) were incubated with or without 50 μg of p4-mAbs for 1 h at room temperature, washed with a fluorescence-activated cell sorting buffer (PBS, 1% FBS, and 2 mM EDTA), and the bacteria were fixed and permeabilized (BioGems). The fixated bacteria were incubated with anti-human IgG-VioBlue (1:50 dilution, Miltenyi Biotec) for 20 min on ice, the unbound antibodies were washed, and the samples were analyzed by cytoflex. For staining of intracellular THP-1-infected H37Ra-mCherry, human monocyte THP-1 cell line^23^ (ATCC) was cultured in RPMI medium (Biological Industries) and differentiated for 24 h by addition of phorbol 12-myristate 13-acetate-(PMA) 160 ng/ml at 37 °C. On the day of infection, bacteria were incubated with 50 μg/ml p4-mAbs and mGO53^20^, used as an isotype control for 30 min at room temperature. The p4-mAbs and bacterial mix, along with anti-human IgG-VioBlue (1:200 dilution, Miltenyi Biotec), were added to 8 × 10^5^ differentiated THP-1 macrophages at MOI 1:10 for 3 h at 37 °C. Following 5 washes with PBS, the cells were incubated with 250 μg/ml Amikacin sulfate salt (Sigma) for 1 h at 37 °C. Cells were washed and dislodged using cell dissociation solution (Biological Industries), and then fixed and permeabilized (BioGems).

### Mycobacterial growth inhibition assay (MGIA)

Blood from healthy volunteer donors was drawn into a CPT vacutainer tube. Blood from the same donor was used for any given set of experiments, to rule out donor-mediated variability. The blood was aspirated into a new 50-ml Falcon tube, and sodium citrate (3.2 g/L) was added as an anticoagulant at a ratio of 1:9 (sodium citrate/blood). Blood was diluted 1:1 with RPMI-1640 (Gibco). A local clinical isolate of pathogenic Mtb, Beijing strain (strain 165^42^), was passed through a 5-µm filter to remove clumps. The OD was checked and diluted with the RPMI-1640 medium. Next, 0.1 ml of the bacterium (10^5^ CFU) was added to 0.9 ml of the diluted blood in 15-ml sterile falcon tubes. A similar protocol was used with the MGIA and BCG infection. An antibody (or PBS) was added to each tube in concentrations as indicated, in triplicates. The tubes were incubated for 96 h at 37 °C in a shaking incubator at 20 rpm. After incubation, the tubes were centrifuged for 10 min at 2000 *g*, and then 8 ml of sterile water was added per tube and the tubes were incubated for 10 min at room temperature. After blood lysis, tubes were spun at 2000 *g* for 10 min, the supernatant discarded, and the pellet resuspended in 1 ml of PBS. The samples were serially diluted and then plated onto OADC-supplemented 7H10 agar plates and incubated for 3 weeks at 37 °C. For the depletion and/or blocking experiments in the MGIA, the experiment was performed as above but with the following modifications^24^. For the T-cell depletion experiments, following a blood draw of 50 µl of the Human CD3 MicroBeads, CD4 or CD8 MicroBeads (Miltenyi Biotec) were added separately into a 2-ml diluted blood sample each and incubated for 30 min at 4 °C. The LS columns (Miltenyi Biotec) were attached to the Midimacs and primed with 3 ml of PBS and 3 ml of RPMI-1640 sequentially. Next, 6 ml of the diluted samples with the beads as above were added to the column. Control blood samples without CD3, CD4, or CD8 microbead incubation were prepared under the same conditions as above. For the receptor blocking experiments, the Fc receptor antibodies anti-human CD32A (clone: 6C4, eBioscience) and anti-human CD16 (clone: 3G8, BioLegend) were used as the blocking antibodies for this assay. In the designated samples, 1 µg of CD32 or 2 µg of CD16 or both were used. For the MHC class II blocking experiments, 4 µg/ml mouse anti-human MHC class II antibody (clone Tu39, BD Biosciences catalog # 555557) was used in the assay.

### Mouse-infection assay

Mice were caged in groups of six in the SPF animal facility. Control mice were caged separately from experimental mice. Monoclonal antibodies (500 µg/mouse, unless indicated otherwise) were injected via the intraperitoneal (IP) route into 6–8-week-old, specific pathogen-free female Balb/c mice (Strain No. 211, purchased from Beijing Vital River Laboratory Animal Technology Co. Ltd., China), 5 h prior to aerosol infection by the Glas-col inhalation exposure system. PBS was injected as a control. Cultures of Mtb strain 165 were diluted to the concentration of 1 × 10^6^ CFU/ml (10 ml) for the aerosol infection. The mice were loaded into the basket of the inhalation exposure system with the lid secured, and the bacterial suspension was loaded into the nebulizer. The set program of the inhalation exposure system was preheating for 15 min and nebulizing for 30 min, cloud decay for 30 min, and decontamination for 15 min. About 24 h following infection, 3 of the PBS control mice were euthanized by cervical dislocation and the lungs were removed and homogenized by MP Fastprep. The actual infection dose delivered to the lungs was verified as between 100 and 200 CFU/mouse for all experiments. The other mice were euthanized by cervical dislocation after 2 weeks, and the lungs were homogenized and plated for the CFU load on OADC-supplemented 7H10 plates as above. The plates were incubated in a 37 °C incubator for around 3 weeks before colonies were counted.

### Complex preparation and crystallization

The Fabs of p4-36 or p4-170 were mixed with PstS1 at a molar ratio of 1:3 at 4 °C. The complex was purified by size-exclusion chromatography with a Superdex 200 Increase 10/300 column running in 150 mM NaCl and 20 mM Tris-HCl, pH 8.0. The peak fractions containing the complex were collected and concentrated for crystallization. The PstS1–Fab p4-36 crystals were grown at 18 °C by using the hanging-drop vapor diffusion method with 1 μl protein (6.2 mg/ml) mixed with 1 μl of reservoir solution containing 0.2 M sodium iodide and 22% (w/v) polyethylene glycol 3350. The PstS1–Fab p4-170 was concentrated to ~10 mg/ml, and the crystals were grown by using a similar method comprising 0.2 M lithium sulfate, 0.1 M Tris, pH 8.5, and 25% (w/v) polyethylene glycol 3350 at 18 °C. Crystals were soaked in a reservoir solution supplemented with 15% glycerol and flash-frozen in liquid nitrogen for data collection.

### Data collection, structure determination, and refinement

The diffraction data were collected on the BL17U beamline at the Shanghai Synchrotron Research Facility. Indexing and integration were performed with the XDS software^43^, followed by scaling and merging with AIMLESS^44^. The structure was determined by molecular replacement using PHASER^45^. Manual building and adjustments of the structures were performed in COOT^46^. The structures were refined by using PHENIX^47^. Data collection and refinement statistics are listed in Supplementary Table 2. Structural analyses of antibody–antigen contacts were assessed through CCP4i^48^ (Supplementary Tables 3 and 4). All structural representations were prepared through the use of the UCSF Chimera^49^.

### PstS1 mutation analysis

Site-directed mutagenesis by PCR was used to introduce point mutations into recombinant PstS1 in the pMALp vector. The PCR products were cleaned with KIT PCR purification (Life Technologies), and the methylated parent DNA was digested using *dpnI* restriction enzyme (NEB). The restriction products were cleaned and transformed into DH5αF^- ^competent cells (Bio-Lab), after which the sequence was validated. The mutated PstS1 proteins were produced similarly to the procedure described above and tested for binding to p4-mAbs in ELISA. Primers for PstS1 mutagenesis are listed in Supplementary Table 5.

### Reporting summary

Further information on research design is available in the Nature Research Reporting Summary linked to this article.

## Supplementary information

Supplementary Information

Reporting Summary

## Data Availability

All data are available in the main text, in the Supplementary Information, or in the Source Data file. Antibody sequence data are available in NCBI database (GenBank accession numbers: p4-36_HC MW355506, p4-36_LC MW355507, p4-163_HC MW355508, p4-163_LC MW355509, p4-170_HC MW355510, and p4-170_LC MW355511). Crystal structures presented in this work are available in RCSB Protein Data Bank (PDB), accession codes 7DM1 (a complex of PstS1 and Fab p4-36) and 7DM2 (a complex of PstS1 and Fab p4-170). The authors declare that all unique materials used are readily available from the authors upon MTA agreement. Source data are provided with this paper.

## Source data

Source Data
