# Supplementary material for: Human antibodies targeting a Mycobacterium transporter protein mediate protection against tuberculosis
Source: Nat Commun. 2021 Jan 27;12:602. doi: 10.1038/s41467-021-20930-0 (PMC7840946; doi:10.1038/s41467-021-20930-0)
Supplement: Supplementary file 1 — Supplementary Information [file 41467_2021_20930_MOESM1_ESM.pdf]

Human Antibodies Targeting a *Mycobacterium* Transporter Protein Mediate Protection Against Tuberculosis

Avia Watson[1,13], Hao Li[2,3,13], Bingting Ma[4,13], Ronen Weiss[1], Daniele Bendayan[5], Lilach Abramovitz[1], Noam Ben-Shalom[1], Michael Mor[1], Erica Pinko[5], Michal Bar Oz[6], Zhenqi Wang[2], Fengjiao Du[7], Yu Lu[7], Jan Rybniker[8,9], Rony Dahan[10], Hairong Huang[11], Daniel Barkan[6], Ye Xiang[4], Babak Javid[2,12] & Natalia T. Freund[1]

Correspondence should be directed to: nfreund@tauex.tau.ac.il (lead contact), bjavid@gmail.com, yxiang@mail.tsinghua.edu.cn

Supplementary Figure 1: Expression of Mtb antigens in *E. coli* fused to maltose binding protein

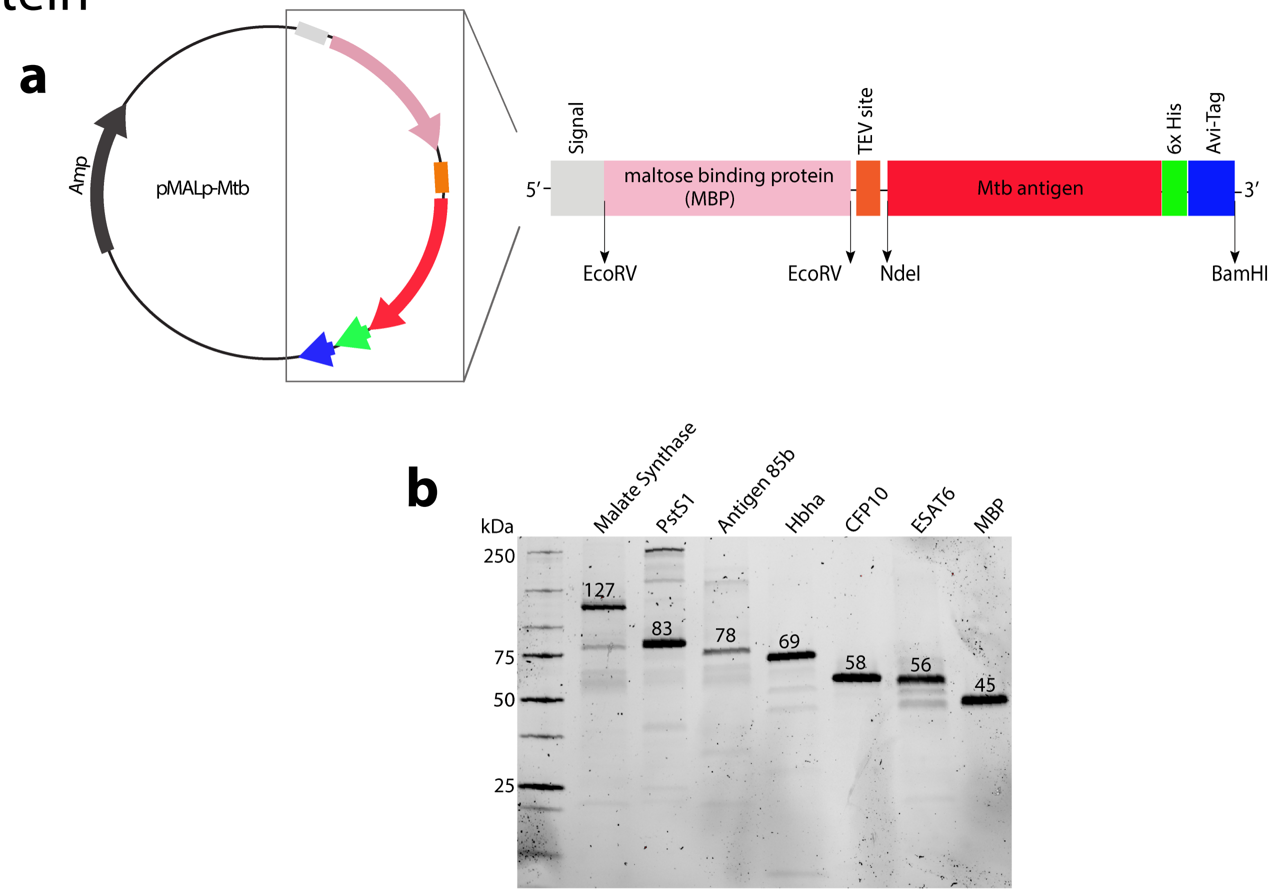

Supplementary Figure 1: Expression of Mtb antigens in *E. coli* fused to maltose binding protein. (a) Schematic map of the pMALp-Mtb plasmid used as backbone for cloning of the selected Mtb antigens. Detailed descriptions of the cloning strategy and of the elements are shown on the right side of the figure. Gray – signal peptide; light pink – maltose binding protein (MBP); orange – TEV cleavage site; red – Mtb antigen, green – 6xHis tag; blue – Avi tag used for BirA biotinylation. **(b)** Protein SDS PAGE stain-free gel showing the different Mtb antigens fused to MBP after Ni affinity chromatography purification. The size of each corresponding MBP-fused protein is indicated. All samples were run on the same gel. The protein gel in representative of at least three independent experiments. Un-cropped gel is provided as a Source Data file.

Supplementary Figure 2: V_H and V_L gene usage in PstS1-sorted memory B cells

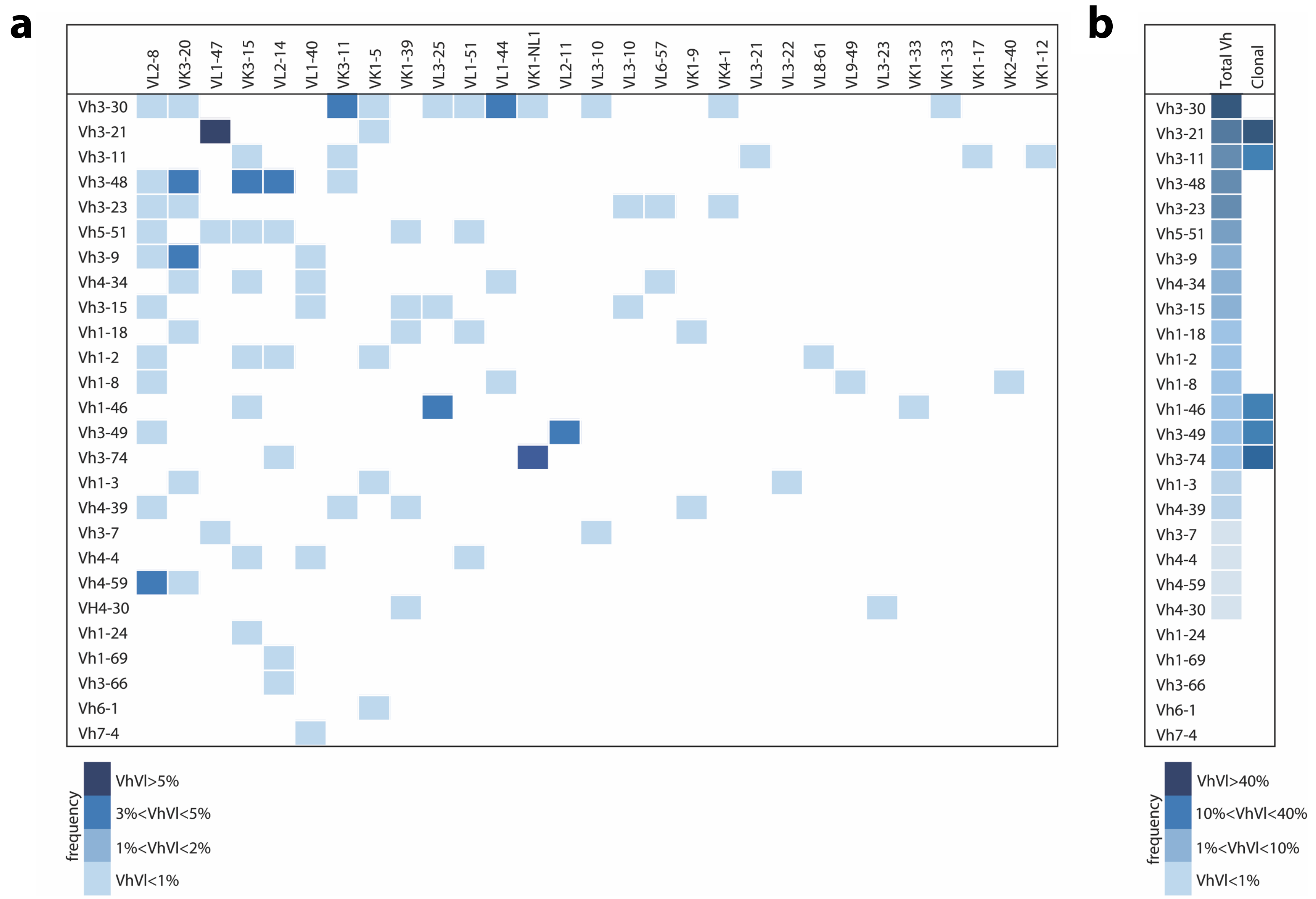

Supplementary Figure 2: VH and VL gene usage in PstS1-sorted memory B cells. **(a)** Gene usage of the different VH and VL paired segments in the PstS1 specific sorted cells. The color code is given in the lower part of the figure. **(b)** VH gene frequency in clonal cells as oppose to single cells.

Supplementary Figure 3: Binding of anti-PstS1 mAbs to PstS1

a

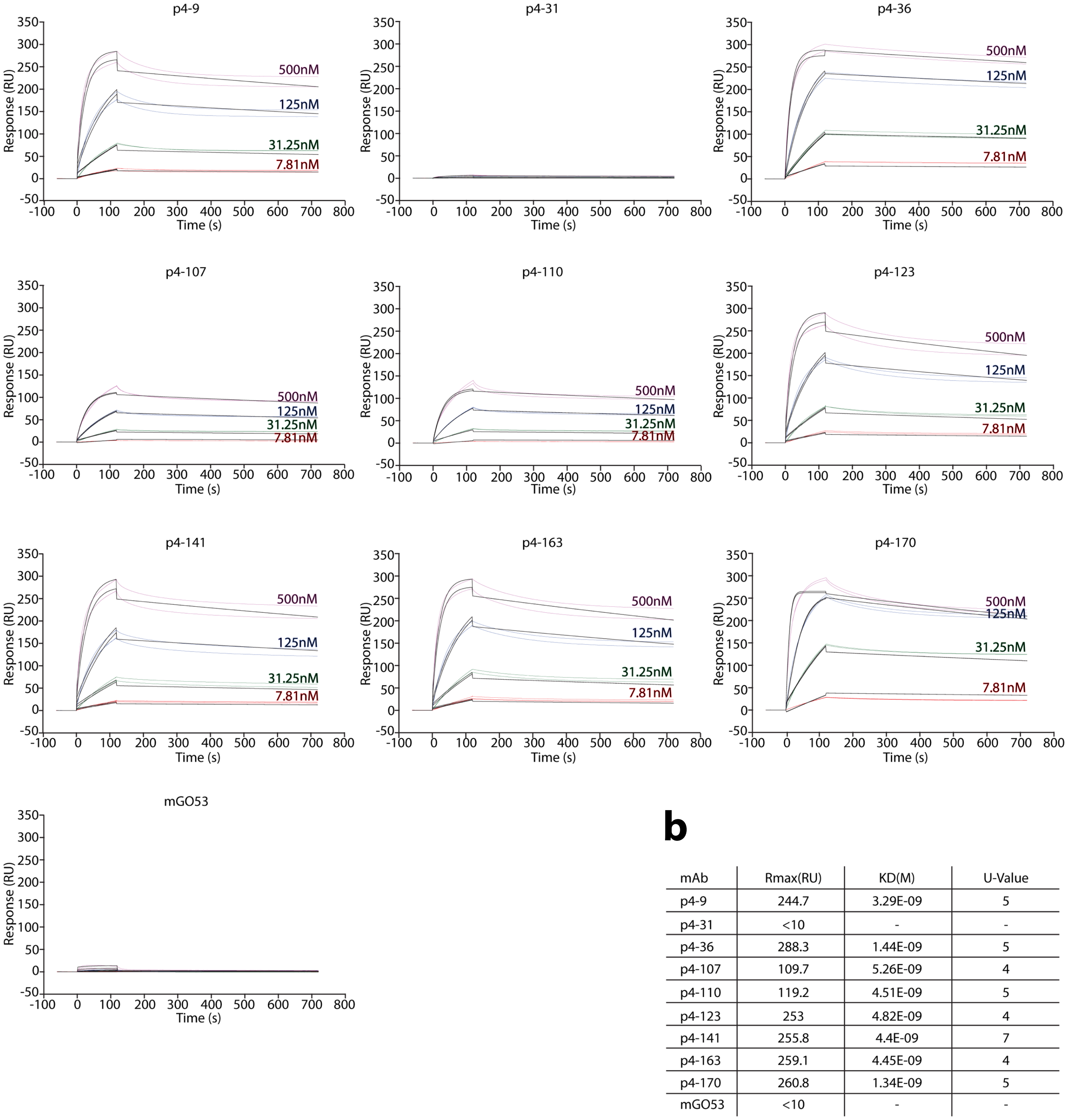

b

mAb	Rmax(RU)	KD(M)	U-Value
p4-9	244.7	3.29E-09	5
p4-31	<10	-	-
p4-36	288.3	1.44E-09	5
p4-107	109.7	5.26E-09	4
p4-110	119.2	4.51E-09	5
p4-123	253	4.82E-09	4
p4-141	255.8	4.4E-09	7
p4-163	259.1	4.45E-09	4
p4-170	260.8	1.34E-09	5
mGO53	<10	-	-

Supplementary Figure 3: Binding of anti-PstS1 mAbs to PstS1. **(a)** SPR sensograms showing the binding of four different concentrations (7.81 nM – red, 31.25 nM – green, 125 nM – blue and 500 nM – pink) of injected PstS1 over immobilized human antibodies. **(b)** Rmax, K_D, and U-value values. Sensograms were fitted to 1:1 binding model using non-linear regression in the BIA evaluation software.

Supplementary Figure 4: Binding of anti-PstS1 mAbs to intact bacteria

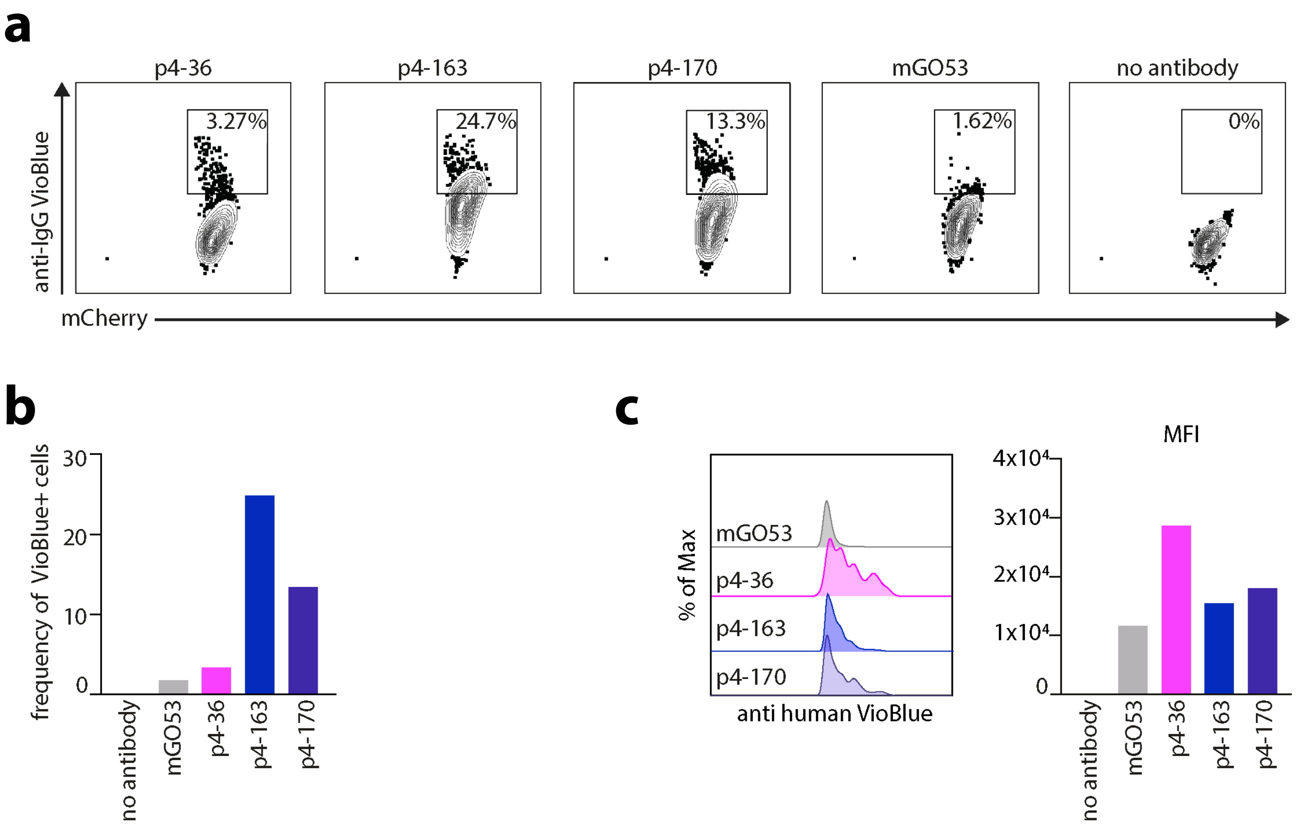

Supplementary Figure 4: Binding of anti-PstS1 mAbs to intact bacteria. **(a)** Reactivity of the whole H37Ra-mCherry bacteria to mAbs, p4-36 (magenta), p4-163 (dark blue), p4-170 (purple), and an isotype control mAb, mGO53 [20] (grey), as determined by flow cytometry. Gating strategy was as follows: All bacteria gated on singlets, followed by mCherry positive bacteria (~93%) followed by anti-IgG positive bacteria. Antibody-bound bacteria was detected by anti-human IgG VioBlue antibody. In each sample 20,000 cells were analyzed. **(b)** Column bar representation of the frequency of mCherry+ VioBlue+ cells showed in **(a)**. **(c)** Histogram (left) and column bars (right) presenting the mean fluorescence intensity (MFI) of mAb-stained H37Ra-mCherry bacteria identified by flow cytometry. All data is representative of three independent experiments.

Supplementary Figure 5: Affinity maturation is necessary for the activity of p4-36 and p4-163 mAbs

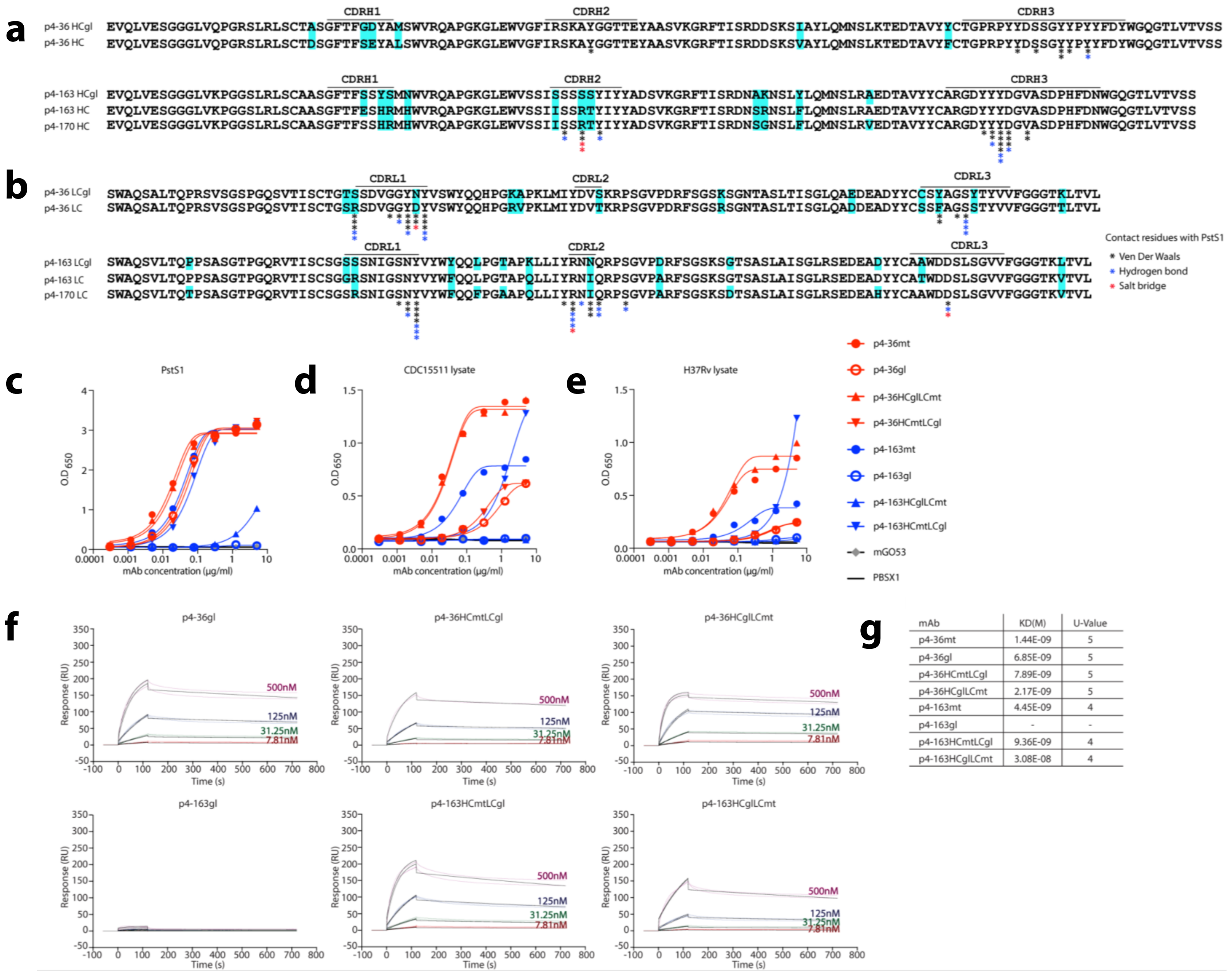

mAb	KD(M)	U-Value
p4-36mt	1.44E-09	5
p4-36gl	6.85E-09	5
p4-36HCmtLCgl	7.89E-09	5
p4-36HCglLCmt	2.17E-09	5
p4-163mt	4.45E-09	4
p4-163gl	-	-
p4-163HCmtLCgl	9.36E-09	4
p4-163HCglLCmt	3.08E-08	4

Supplementary Figure 5: Affinity maturation is necessary for the activity of p4-36 and p4-163 mAbs. **(a)** and **(b)** amino acid sequence alignment of the heavy chains and light chains of mAbs p4-36, p4-163, and p4-170, and their predicted germline versions, respectively. The alignment of p4-36 is shown in the top panel, and the alignment of Clone 1 antibodies (p4-163 and p4-170) is in the bottom panel. The positions that are different from the germline are colored in cyan. Residues that make contact with PstS1 are indicated underneath by black, blue and red asterisks, and correspond to Van der Waals, Hydrogen and salt bridges, respectively. **(c), (d)** and **(e)** Binding by ELISA of the mature (mt) and the predicted germline (gl) versions of p4-36 (red) and p4-163 (blue), as well as their chimeras (mtHCglLC and glHCmtLC) to recombinant PstS1, Mtb-CDC1551 lysate, and Mtb-H37Rv lysate, respectively. mGO53 [20] serves as an isotype control. **(f)** SPR sensorgams showing the binding of four different concentrations of PstS1 (7.81 nM – red, 31.25 nM – green, 125 nM – blue and 500 nM – pink) to immobilized germline versions of p4-36 and p4-163 and their chimeras. Upper three panels from left to right: fully germline mAb p4-36 (p4-36gl), p4-36HCmtLCgl and p4-36HCglLCmt. Lower three panels from left to right: fully germline mAb p4-163 (p4-163gl) and p4-163HCmtLCgl and p4-163HCglLCmt. **(g)** A table summarizing the K_D and U-value values of the germline and germline-mature chimera antibodies. The mature p4-36 and p4-163 are presented for comparison (data from Supplementary, Fig.3). Sensograms were fitted to 1:1 binding model using non-linear regression in the BIA evaluation software.

Supplementary Figure 6: Asymmetric unit of the PstS1-Fab p4-36 complex

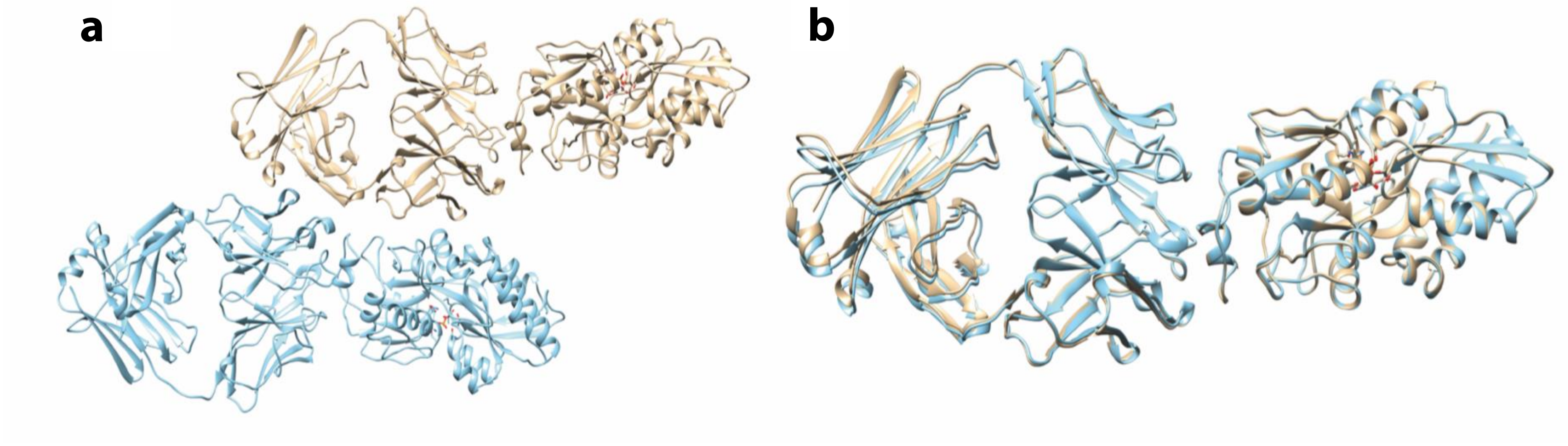

Supplementary Figure 6: Asymmetric unit of the PstS1-Fab p4-36 complex. **(a)** Ribbon diagrams of two PstS1-Fab p4-36 heterodimers in the asymmetric unit of the crystal. The two heterodimers are colored cyan and yellow, respectively. **(b)** Structural superimposition of two PstS1-Fab p4-36 heterodimers in the asymmetric unit showing minor differences between the constant regions of the bound Fabs.

Supplementary Figure 7: Comparisons of the epitopes recognized by p4-36 and p4-170

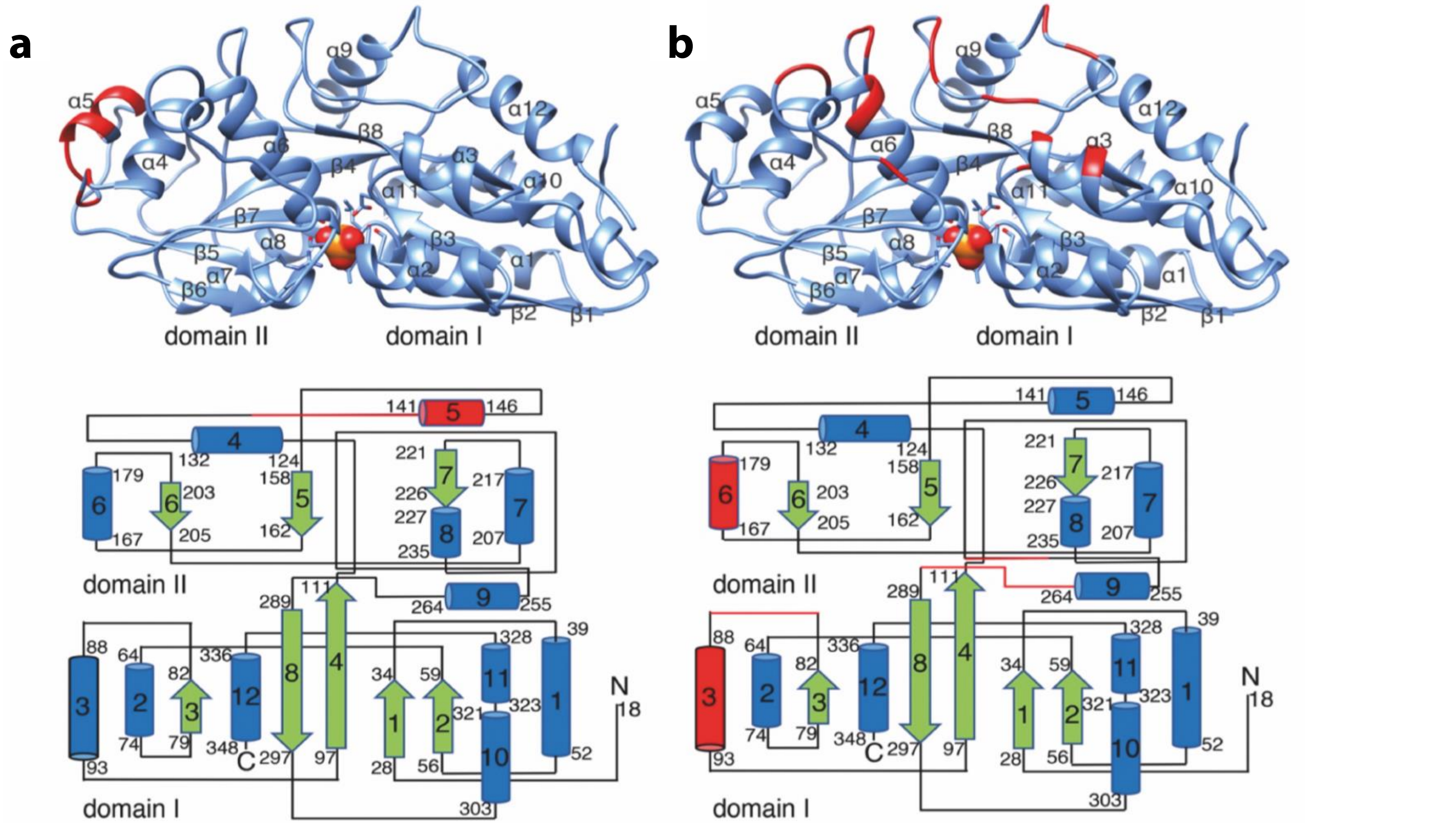

Supplementary Figure 7: Comparisons of the epitopes recognized by p4-36 and p4-170. (a) Ribbon (upper panel) and topology (lower panel) diagrams of PstS1 showing the epitope recognized by p4-36. The structural segments constituting the epitope are colored red. **(b)** Ribbon (upper panel) and topology (lower panel) diagrams of PstS1 showing the epitope recognized by p4-170. The structural segments constituting the epitope are colored red. Strands are represented as light green arrows and helixes are represented as blue cylinders in the topology diagrams.

Supplementary Figure 8: Residues and CDRs of p4-36 and p4-170 involved in recognizing PstS1

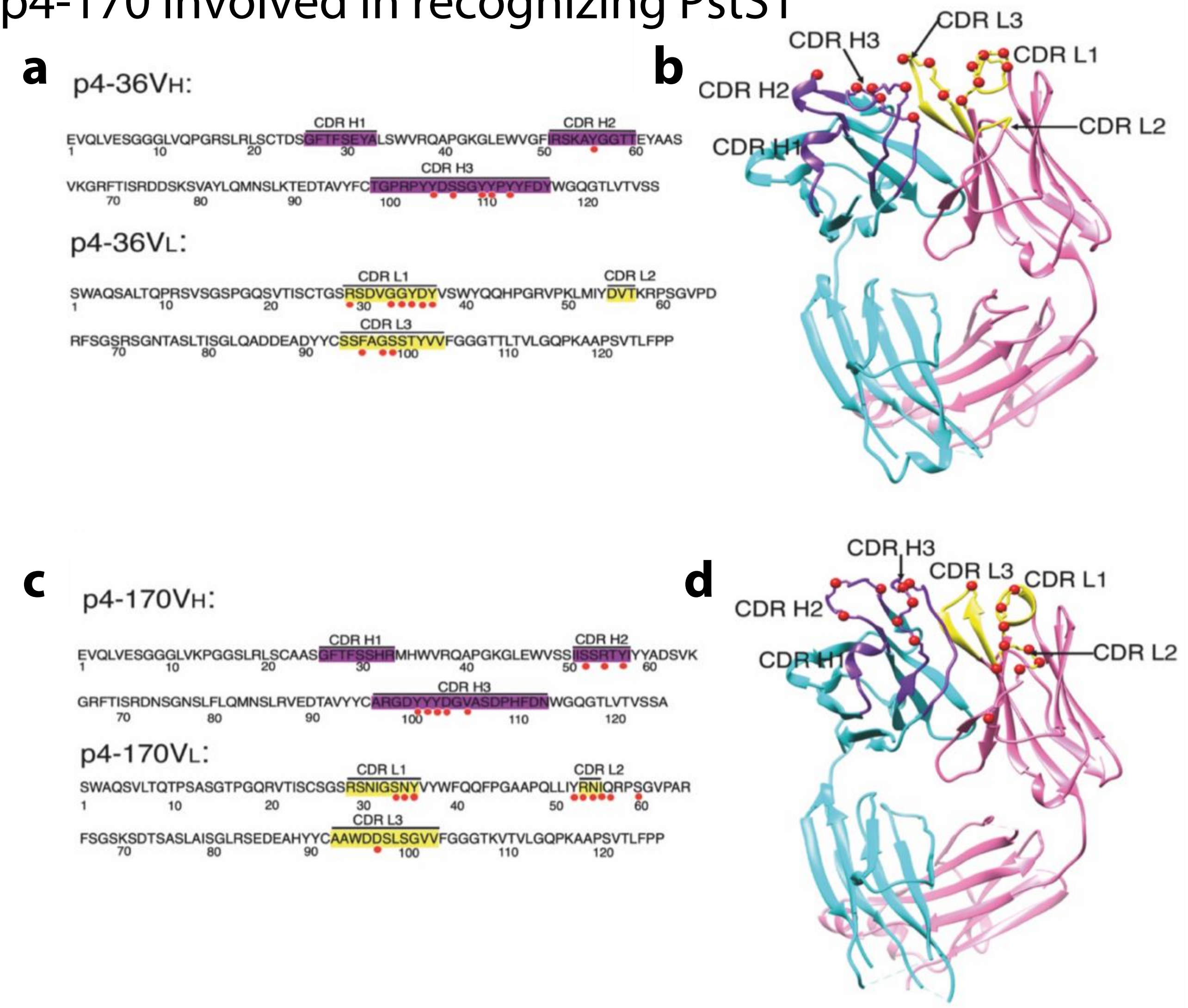

Supplementary Figure 8: Residues and CDRs of p4-36 and p4-170 involved in recognizing PstS1. (a) Amino acid sequences of the p4-36 heavy chain variable region (VH) and light chain variable region (VL). The CDR regions in VH and VL are highlighted in purple and yellow, respectively. Residues in contacts with PstS1 are marked with red dots at the bottom of the sequence. **(b)** Ribbon diagrams of Fab p4-36 showing the CDRs and residues involved in binding PstS1. The CDRs of the heavy chain (cyan) and the light chain (hot pink) are colored purple and yellow, respectively. Positions of the residues involved in binding PstS1 are indicated by red balls. **(c)** Amino acid sequences of the p4-170 heavy chain variable region (VH) and light chain variable region (VL). The CDR regions in VH and VL are highlighted in purple and yellow, respectively. Residues in contacts with PstS1 are marked with red dots at the bottom of the sequence. **(d)** Ribbon diagrams of Fab p4-170 showing the CDRs and residues involved in binding PstS1. The CDRs of the heavy chain (cyan) and the light chain (hot pink) are colored purple and yellow, respectively. Positions of the residues involved in binding PstS1 are indicated by red balls.

Supplementary Figure 9:
Anti-PstS1 mAbs, p4-36, p4-163 and p4-170 inhibit Mtb in MGIA

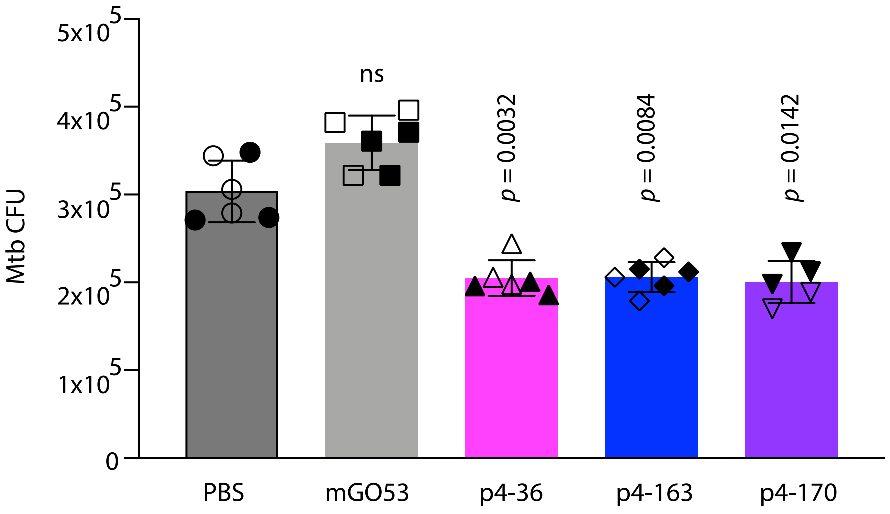

Supplementary Figure 9: Anti-PstS1 mAbs p4-36, p4-163 and p4-170 inhibit Mtb in MGIA. Activity of anti-PstS1 mAbs p4-36 (magenta), p4-163 (blue) and p4-170 (purple) at 5 µg/ml concentration in MGIA after 96 hours of infection with pathogenic Mtb. Black and clear shapes correspond to two independent experiments. Error bar represent mean±SD. Significance was determined for black shapes, n=3 biological repetitions using One-way ANOVA with Tukey's multiple comparison test relatively to PBS.

Supplementary Table 1. Patient clinical data

Patient	Sample ID	Group	Sex	Age	Plasma collection date	Resistance to treatment	Medical backgound
P.1	109001	Active TB	Male	60	25/09/17	DS	Hep B positive
P.2	109002	Active TB	Male	47	25/09/17	Non-tuberculous mycobacteria sensitive	Hep A and B positive
P.3	109003	Active TB	Male	33	25/09/17	DS	Diabetic, Cirrhosis, Chronic pancreatitis
P.4	109004	Active TB	Male	35	25/09/17	DS	
P.5	109005	Active TB	Male	46	25/09/17	MDR	
P.6	109006	Active TB	Female	27	25/09/17	DS	
P.7	109007	Active TB	Male	33	26/09/17	DS	HCV positive
P.8	109008	Active TB	Male	26	26/09/17	DS	
P.9	109009	Active TB	Male	24	26/09/17	DS	
P.10	109010	Active TB	Male	28	26/09/17	DS	
P.11	109011	Active TB	Female	37	26/09/17	DS	
P.12	109012	Active TB	Male	29	27/09/17	DS	Hep B positive
P.13	109013	Active TB	Male	32	27/09/17	DS	
P.14	109014	Active TB	Female	24	19/10/17	DS	
P.15	109015	Active TB	Female	45	19/10/17	Rifampicin DR	Diabetic
P.17	109017	Active TB	Male	27	19/10/17	DS	
P.19	109019	Active TB	Male	25	19/10/17	DS	
P.20	109020	Active TB	Male	16	19/10/17	DS	
P.21	109021	Active TB	Male	26	19/10/17	DS	
P.22	109022	Active TB	Female	42	26/11/17	DS	Diabetic
P.24	109024	Active TB	Female	34	26/11/17	DS	HIV positive
P.25	109025	Active TB	Female	39	26/11/17	DS	
P.26	109026	Active TB	Female	24	26/11/17	DS	
P.27	109027	Active TB	Male	25	28/11/17	DS	
P.28	109028	Active TB	Male	36	28/11/17	DS	
P.29	109029	Active TB	Male	45	28/11/17	Streptomycin DR	

DS	Drug sensitive
DR	Drug resistance
MDR	Multi drug resistance

Supplementary Table 2. Data collection and refinement statistics for PstS1-Fab complex

Related to Figure 4.

	PstS1-Fab36	PstS1-Fab170
**Data collection**		
Wavelength(Å)	0.979	0.979
Resolution(Å)	54.07-2.1 (2.175-2.1)	28.08-2.4 (2.486-2.4)
Space group	P 1 21 1	P 1
Unit cell	92.6, 78.1, 132.6	56.4, 56.7, 65.8
	90.0, 90.4, 90.0	102.3, 110.3, 93.2
Unique reflections	106547 (10692)	27948 (2640)
Completeness (%)	96.18 (96.88)	96.38 (90.59)
Rsym or R-merge	0.0612 (0.264)	0.04484 (0.166)
Redundancy	3.2 (2.3)	2.3 (1.8)
I/σ (I)	11.57 (2.80)	15.14 (4.92)
**Statistics for Refinement**		
R-work	0.1869 (0.2354)	0.1703 (0.2071)
R-free	0.2299 (0.2910)	0.2161(0.2587)
R.m.s.d		
Bond(degree)	0.86	0.69
Length(Å)	0.007	0.005
Ramachandran plot		
Favored region (%)	97.04	97.6
Allowed region (%)	2.83	2.27
Outliers (%)	0.13	0.13

Supplementary Table 3. Fab p4-36/PstS1 interface

Related to Figure 4.

Van der Waals contacts[a]		Direct hydrogen bonds[b]			Salt-bridges[b]		
PstS1	Fab P4-36	PstS1	Fab P4-36	Distance (Å)	PstS1	Fab P4-36	Distance (Å)
THR^{A134}	ASP^{L36}(6)						
LYS^{A136}	ARG^{L29}(4)	LYS^{A136}[O]	ARG^{L29}[NH1]	2.92	LYS^{A136}[NZ]	ASP^{L36}[OD1]	2.72
	GLY^{L33}(2)						
	ASP^{L36}(1)						
THR^{A137}	ARG^{L29}(3)	THR^{A137}[OG1]	ARG^{L29}[NH2]	2.46			
	GLY^{L34}(3)						
	TYR^{L35}(3)	THR^{A137}[OG1]	GLY^{L34}[O]	2.75			
ASP^{A139}	TYR^{L35}(4)	ASP^{A139}[OD1]	TYR^{L35}[OH]	2.58			
	PHE^{L96}(2)						
	GLY^{L98}(2)	ASP^{A139}[OD2]	SER^{L99}[N]	3.04			
	SER^{L99}(3)	ASP^{A139}[OD2]	SER^{L99}[OG]	3.34			
ASP^{A140}	TYR^{L35}(2)	ASP^{A140}[OD1]	TYR^{L37}[OH]	2.55			
	TYR^{L37}(7)						
PRO^{A141}	TYR^{L37}(3)						
	PHE^{L96}(2)						
	TYR^{H111}(2)						
	TYR^{H113}(5)						
GLN^{A142}	TYR^{L37}(2)	GLN^{A142}[NE2]	TYR^{L37}[OH]	3.02			
	TYR^{H105}(4)	GLN^{A142}[N]	TYR^{H113}[OH]	3.73			
ALA^{A144}	TYR^{H110}(5)						
ALA^{A145}	TYR^{H56}(1)						
	SER^{H107}(1)						
	TYR^{H110}(4)						
		LEU^{A152}[O]	SER^{L99}[OG]	3.84			
ALA^{A156}	ARG^{L29}(2)						

[a] Van der Waals contacts (# in parentheses) have interatomic distance ≤ 3.9 Å and defined by CCP4i.

[b] Putative H-bonds and salt-bridges defined by PISA.

Supplementary Table 4. Fab p4-170/PstS1 interface

Related to Figure 4.

Van der Waals contacts[a]		Direct hydrogen bonds[b]			Salt-bridges[b]		
PstS1	Fab P4-170	PstS1	Fab P4-170	Distance (Å)	PstS1	Fab P4-170	Distance (Å)
TYR^{A85}	TYR^{H103}(3)	TYR^{A85}[OH]	TYR^{H103}[O]	2.5			
	ASP^{H104}(1)						
GLU^{A88}	SER^{H53}(3)	GLU^{A88}[OE1]	SER^{H53}[OG]	2.85	GLU^{A88}[OE1]	ARG^{H55}[NE]	2.54
	ARG^{H55}(2)						
	TYR^{H102}(5)	GLU^{A88}[OE2]	TYR^{H102}[OH]	2.55	GLU^{A88}[OE1]	ARG^{H55}[NH1]	3.2
ALA^{A92}	ARG^{H55}(1)						
		LYS^{A125}[NZ]	GLN^{L57}[OE1]	3.09			
		GLN^{A174}[NE2]	TYR^{H103}[O]	3.58			
SER^{A177}	TYR^{H103}(3)						
LYS^{A178}	TYR^{H103}(9)						
PRO^{A181}	TYR^{L53}(1)						
GLU^{A182}	SER^{L60}(5)	GLU^{A182}[OE1]	SER^{L60}[N]	2.96			
PHE^{A190}	TYR^{H101}(1)						
	TYR^{H102}(2)						
	TYR^{H103}(1)						
SER^{A246}	TYR^{L36}(3)	SER^{A246}[O]	GLN^{L57}[NE2]	3.05			
	ARG^{L54}(3)						
	ILE^{L56}(2)	SER^{A246}[OG]	ARG^{L54}[NE]	3.28			
	GLN^{L57}(2)	SER^{A246}[OG]	ARG^{L54}[NH2]	3.42			
SER^{A247}	ILE^{L56}(2)	SER^{A247}[OG]	ASN^{L55}[ND2]	2.85			
GLY^{A248}	ILE^{L56}(2)						
		SER^{A267}[OG]	ASP^{L97}[OD2]	3.63			
LYS^{A268}	VAL^{H106}(1)	LYS^{A268}[NZ]	ASN^{L35}[OD1]	2.82	LYS^{A268}[NZ]	ASP^{L97}[OD1]	3.33
	ASN^{L35}(2)						
	ASP^{L97}(2)						
PRO^{A270}	TYR^{H57}(5)						
		ALA^{A271}[N]	TYR^{H57}[OH]	3.15			
ILE^{A275}	ASP^{H104}(3)						
	VAL^{H106}(2)						
SER^{A276}	ASP^{H104}(4)	SER^{A276}[N]	ASP^{H104}[OD2]	2.84			
ASP^{A279}	TYR^{L36}(4)	ASP^{A279}[O]	TYR^{L36}[OH]	2.56	ASP^{A279}[OD2]	ARG^{L54}[NH2]	2.83
	ARG^{L54}(3)	ASP^{A279}[O]	ARG^{L54}[NH2]	3.11			
		ASP^{A279}[OD1]	TYR^{L36}[OH]	3.36			
GLY^{A280}	TYR^{L36}(3)	GLY^{A280}[O]	TYR^{L36}[OH]	2.83			
PRO^{A281}	SER^{L34}(2)						
	ASN^{L35}(1)						
	TYR^{L36}(6)						

[a] Van der Waals contacts (# in parentheses) have interatomic distance ≤ 3.9 Å and defined by CCP4i.

[b] Putative H-bonds and salt-bridges defined by PISA.

Supplementary Table 5: Primers for PstS1 mutagenesis

Related to Figure 4e.

Primer name	Sequence
PstS1_sense_K136E	GGCGGCGATGTATCAGGGCACCATCgAAACCTGGGACGATCCGCAAATTG
PstS1_antisense_K136E	CAATTTGCGGATCGTCCCAGGTTTcGATGGTGCCCTGATACATCGCCGCC
PstS1_sense_D139A	CAGGGCACCATCAAAACCTGGGcCGATCCGCAAATTGCGGCGCTGAACCC
PstS1_antisense_D139A	GGGTTCAGCGCCGCAATTTGCGGATCGgCCCAGGTTTTGATGGTGCCCTG
PstS1_sense_D140A	GTATCAGGGCACCATCAAAACCTGGGcCGATCCGCAAATTGCGGCGCTGAAC
PstS1_antisense_D140A	GTTCAGCGCCGCAATTTGCGGATCGgCCCAGGTTTTGATGGTGCCCTGATAC
PstS1_sense_S246G	CAGCTGGGTAACgGCAGCGGCAACTTCCTGCTGCCGGATG
PstS1_antisense_S246G	CATCCGGCAGCAGGAAGTTGCCGCTGCcGTTACCCAGCTG
PstS1_sense_K268E	GTTTTGCGAGCgAGACCCCGGCGAACCAGGCGATCAGCATGATTGATGGTC
PstS1_antisense_K268E	GACCATCAATCATGCTGATCGCCTGGTTCGCCGGGGTCTcGCTCGCAAAAC
PstS1_sense_D279A	GATCAGCATGATTGcTGGTCCGGCGCCGGATGGCTACCCGATCATTAACTAC
PstS1_antisense_D279A	GTAGTTAATGATCGGGTAGCCATCCGGCGCCGGACCAgCAATCATGCTGAT

*Red highlighted lowercase indicates the mutagenesis site and replecment codon
