# Supplementary material for: Human antibodies targeting a Mycobacterium transporter protein mediate protection against tuberculosis
Source: Nat Commun. 2021 Jan 27;12:602. doi: 10.1038/s41467-021-20930-0 (PMC7840946; doi:10.1038/s41467-021-20930-0)
Supplement: Supplementary file 2 — Reporting Summary [file 41467_2021_20930_MOESM2_ESM.pdf]

Corresponding author(s): 20-17730A

Last updated by author(s): Dec 10, 2020

Reporting Summary

Nature Research wishes to improve the reproducibility of the work that we publish. This form provides structure for consistency and transparency in reporting. For further information on Nature Research policies, see our Editorial Policies and the Editorial Policy Checklist.

Statistics

For all statistical analyses, confirm that the following items are present in the figure legend, table legend, main text, or Methods section.

n/a	Confirmed	
☐	☒	The exact sample size (*n*) for each experimental group/condition, given as a discrete number and unit of measurement
☐	☒	A statement on whether measurements were taken from distinct samples or whether the same sample was measured repeatedly
☐	☒	The statistical test(s) used AND whether they are one- or two-sided<br>*Only common tests should be described solely by name; describe more complex techniques in the Methods section.*
☐	☒	A description of all covariates tested
☐	☒	A description of any assumptions or corrections, such as tests of normality and adjustment for multiple comparisons
☐	☒	A full description of the statistical parameters including central tendency (e.g. means) or other basic estimates (e.g. regression coefficient) AND variation (e.g. standard deviation) or associated estimates of uncertainty (e.g. confidence intervals)
☐	☒	For null hypothesis testing, the test statistic (e.g. *F*, *t*, *r*) with confidence intervals, effect sizes, degrees of freedom and *P* value noted<br>*Give P values as exact values whenever suitable.*
☒	☐	For Bayesian analysis, information on the choice of priors and Markov chain Monte Carlo settings
☐	☒	For hierarchical and complex designs, identification of the appropriate level for tests and full reporting of outcomes
☒	☐	Estimates of effect sizes (e.g. Cohen's *d*, Pearson's *r*), indicating how they were calculated

Our web collection on statistics for biologists contains articles on many of the points above.

Software and code

Policy information about availability of computer code

Data collection	IMGT database
Data analysis	we used an in house program for aligning the B cell sequences from patient 004 to the IMGT database

For manuscripts utilizing custom algorithms or software that are central to the research but not yet described in published literature, software must be made available to editors and reviewers. We strongly encourage code deposition in a community repository (e.g. GitHub). See the Nature Research guidelines for submitting code & software for further information.

Data

Policy information about availability of data

All manuscripts must include a data availability statement. This statement should provide the following information, where applicable:

- Accession codes, unique identifiers, or web links for publicly available datasets
- A list of figures that have associated raw data
- A description of any restrictions on data availability

All data is available in the main text, in the Supplementary Data, Materials or in the Source Data files. Antibody sequence data has been deposited in the NCBI database under Submission 2406654. Crystal structures presented in this work has been deposited in the RCSB Protein Data Bank (PDB) and are available with accession codes 7DM1 (complex of PstS1 and Fab p4-36) and 7DM2 (complex of PstS1 and Fab p4-170). The authors declare that all unique materials used are readily available from the authors upon MTA agreement. Source data are provided with this paper.

Field-specific reporting

Please select the one below that is the best fit for your research. If you are not sure, read the appropriate sections before making your selection.

- [x] Life sciences
- [ ] Behavioural & social sciences
- [ ] Ecological, evolutionary & environmental sciences

For a reference copy of the document with all sections, see nature.com/documents/nr-reporting-summary-flat.pdf

Life sciences study design

All studies must disclose on these points even when the disclosure is negative.

Sample size	For human studies: 26 patients with active Tuberculosis, gender was added. Based on previous literature we expected about 0.5-1% responders to each antigen, therefore we wished to recruit between 20-30 infected subjects. For mouse studies: each experiment included Balb/c 6 mice based on previous work. The experiments we conducted three times unless indicated otherwise.
Data exclusions	No data was excluded
Replication	All mouse experiment were conducted three times unless indicated otherwise. All the mice experiments were successful. All The ELISA experiments testing the human sera for binding to MTb lysates or antigens were conducted at least three times for every patient with every lysate/antigen. For H37rv lysate and CDC1551 ELISA's were done twice.
Randomization	all samples were randolmy labeled, and only after the data was available the samples were de-coded
Blinding	The antibodies were isolated by the laboratory in Tel Aviv and sent to Beijing without identification. The investigators in Beijing tested the antibodies for neutralization blindly and reported the results.

Reporting for specific materials, systems and methods

We require information from authors about some types of materials, experimental systems and methods used in many studies. Here, indicate whether each material, system or method listed is relevant to your study. If you are not sure if a list item applies to your research, read the appropriate section before selecting a response.

Materials & experimental systems

n/a	Involved in the study	
☐	☒	Antibodies
☐	☒	Eukaryotic cell lines
☒	☐	Palaeontology and archaeology
☐	☒	Animals and other organisms
☐	☒	Human research participants
☒	☐	Clinical data
☒	☐	Dual use research of concern

Methods

n/a	Involved in the study	
☒	☐	ChIP-seq
☐	☒	Flow cytometry
☒	☐	MRI-based neuroimaging

Antibodies

Antibodies used	anti human igg-hrp, jackson, catalog: 109-035-088, lot: 132329; anti-human igg vio-blue, miltenyi, catalog: 130-119-881, clone: is11-3b2.2.3, lot: 5191212216; cd19anti-human vioblue, miltenyi, catalog: 130-113-172, clone:lt19, lot: 5200700754; anti-igg apc, miltenyi, catalog: 130-119-772, clone: is11-3b2.2.3, lot: 5200400050
Validation	All antibodies used for FACS were used separately to stain cells and validate their specificity and activity. In ELISA we tested the binding of the secondary antibody to MTb lysates as well as antigens. human IgG was coated on the plate as a positive control. Cross reactivity to other IgGs was tested and found to be negligible

Eukaryotic cell lines

Policy information about cell lines

Cell line source(s)	THP-1 macrophages
Authentication	cells are from ATCC material resource
Mycoplasma contamination	all cell lines were tested negative for mycoplasma

Commonly misidentified lines (See ICLAC register)	none

Animals and other organisms

Policy information about studies involving animals; ARRIVE guidelines recommended for reporting animal research

Laboratory animals	Specific pathogen-free female BALB/c mice (Strain No.211) were purchased from Beijing Vital River Laboratory Animal Technology Co. Ltd (Beijing, China).
Wild animals	no
Field-collected samples	no
Ethics oversight	Chinese Association for Laboratory Animal Sciences, and approved by the institutional ethical Committee (IEC) of Peking Union Medical College

Note that full information on the approval of the study protocol must also be provided in the manuscript.

Human research participants

Policy information about studies involving human research participants

Population characteristics	26 in-patients with Active tuberculosis disease between the ages 18-65. All patients received standard treatment, recovered, and were eventually discharged. 18 males and 8 females
Recruitment	All participants were recruited in Shmuel Harofe hospital and signed a consent form, exclusion criteria were children, and insufficient blood hemoglobin levels.
Ethics oversight	Tel Aviv University Institutional Review Board (IRB) approved all studies involving patient enrollment, sample collection, and clinical follow-up. Donors provided written informed consent prior to participating in this study, and The Tel Aviv University and Shmuel Harofe Hosptial Institutional Review Boards approved all studies involving patient enrollment, sample collection, and clinical follow-up (protocols number 33.18. and 0058).

Note that full information on the approval of the study protocol must also be provided in the manuscript.

Flow Cytometry

Plots

Confirm that:

- [x] The axis labels state the marker and fluorochrome used (e.g. CD4-FITC).
- [x] The axis scales are clearly visible. Include numbers along axes only for bottom left plot of group (a 'group' is an analysis of identical markers).
- [x] All plots are contour plots with outliers or pseudocolor plots.
- [x] A numerical value for number of cells or percentage (with statistics) is provided.

Methodology

Sample preparation	Frozen PBMCs were thawed and immediately stained
Instrument	FACS ARIA III
Software	Analysis by Flowjo
Cell population abundance	After thawing the PBMCs from the donor, B cell enrichment with CD20 magnetic beads was carried out, followed with over 80% B cells, 3.6% of which were IgG expressing cells. Within this population 0.5% were positive to PstS1 bait.
Gating strategy	We gated on live cells, singlets, followed by gating on CD19+IgG+ population (comprising 3.6% of the live singlets cells). Next, we gated on PstS1 positive cells that were single-cell sorted.

- [ ] Tick this box to confirm that a figure exemplifying the gating strategy is provided in the Supplementary Information.

April 2020
